# Inhibiting ERK dimerization ameliorates BRAF-driven anaplastic thyroid cancer

**DOI:** 10.1007/s00018-022-04530-9

**Published:** 2022-09-03

**Authors:** Miguel A. Zaballos, Adrián Acuña-Ruiz, Marta Morante, Garcilaso Riesco-Eizaguirre, Piero Crespo, Pilar Santisteban

**Affiliations:** 1 Instituto de Investigaciones Biomédicas “Alberto Sols”, Consejo Superior de Investigaciones Científicas and Universidad Autónoma de Madrid (CSIC-UAM), 28029 Madrid, Spain; 2 Centro de Investigación Biomédica en Red de Cáncer (CIBERONC), Instituto de Salud Carlos III (ISCIII), 28029 Madrid, Spain; 3 Departamento de Endocrinología y Nutrición, Hospital Universitario de Móstoles, 28935 Madrid, Spain; 4 Present Address: Instituto de Biomedicina y Biotecnología de Cantabria (IBBTEC), Consejo Superior de Investigaciones Científicas (CSIC)-Universidad de Cantabria. Santander, 39011 Cantabria, Spain; 5 Grupo de Endocrinología Molecular, Facultad de Medicina, Universidad Francisco de Vitoria, 28223 Madrid, Spain

**Keywords:** Thyroid cancer, DEL-22379, ERK dimerization, BRAF, RAS

## Abstract

**Background:**

RAS-to-ERK signaling is crucial for the onset and progression of advanced thyroid carcinoma, and blocking ERK dimerization provides a therapeutic benefit in several human carcinomas. Here we analyzed the effects of DEL-22379, a relatively specific ERK dimerization inhibitor, on the activation of the RAS-to-ERK signaling cascade and on tumor-related processes in vitro and in vivo.

**Methods:**

We used a panel of four human anaplastic thyroid carcinoma (ATC) cell lines harboring BRAF or RAS mutations to analyze ERK dynamics and tumor-specific characteristics. We also assessed the impact of DEL-22379 on the transcriptional landscape of ATC cell lines using RNA-sequencing and evaluated its therapeutic efficacy in an orthotopic mouse model of ATC.

**Results:**

DEL-22379 impaired upstream ERK activation in BRAF- but not RAS-mutant cells. Cell viability and metastasis-related processes were attenuated by DEL-22379 treatment, but mostly in BRAF-mutant cells, whereas in vivo tumor growth and dissemination were strongly reduced for BRAF-mutant cells and mildly reduced for RAS-mutant cells. Transcriptomics analyses indicated that DEL-22379 modulated the transcriptional landscape of BRAF- and RAS-mutant cells in opposite directions.

**Conclusions:**

Our findings establish that BRAF- and RAS-mutant thyroid cells respond differentially to DEL-22379, which cannot be explained by the previously described mechanism of action of the inhibitor. Nonetheless, DEL-22379 demonstrated significant anti-tumor effects against BRAF-mutant cells in vivo with an apparent lack of toxicity, making it an interesting candidate for the development of combinatorial treatments. Our data underscore the differences elicited by the specific driver mutation for thyroid cancer onset and progression, which should be considered for experimental and clinical approaches.

**Supplementary Information:**

The online version contains supplementary material available at 10.1007/s00018-022-04530-9.

## Background

The incidence of thyroid cancer has steadily risen in the past 4 decades [1]. Approximately 90% of all carcinomas derived from the thyroid gland originate from the epithelial follicular cells that form thyroid follicles––the structural and functional unit of the thyroid gland [2]. Well-differentiated thyroid carcinoma (WDTC) is the most common type of thyroid cancer and includes papillary thyroid carcinoma (PTC) [3], comprising 80–85% of all thyroid cancer cases, and follicular thyroid carcinoma (FTC) [4], encompassing 10–15%. Both PTC and FTC are indolent carcinomas that retain a differentiated state, and patients generally have a very good prognosis (5-year survival rate > 90%) following treatment, which can include surgical removal of the gland followed by radioiodide administration to eliminate remnant tissue [5]. A small subset of WDTC progresses to undifferentiated and very aggressive carcinoma, which is accompanied by metastatic events and high mortality rates, and for which no effective treatment yet exists. The most aggressive type is anaplastic thyroid carcinoma (ATC) [6], a rare carcinoma (1–2% incidence) with a dismal outcome and mean survival of 6 months from diagnosis. Poorly differentiated thyroid carcinoma (PDTC) [7] is thought to be an intermediate stage between WDTC and ATC, although in terms of aggressiveness and survival it is more similar to ATC [8].

The key genetic events responsible for thyroid cancer are mutations in BRAF and RAS oncogenes, which are components of the RAS-to-ERK signaling pathway. BRAF and RAS mutations are mutually exclusive and either mutation is capable of sustaining thyroid tumorigenesis [9]; however, the initial driver mutation confers very different tumor characteristics. For example, PTC is enriched for BRAF mutations, presents with a papillary morphology and disseminates through lymph nodes, whereas FTC is enriched for RAS mutations, retains a follicular morphology and disseminates through blood vessels. Moreover, both subtypes display different patterns of RNA expression and DNA methylation, and respond differentially to therapeutic approaches [10–12].

BRAF is the most frequently mutated gene in thyroid tumors and has been the target of several clinical trials, including for advanced forms of the disease that are refractory to conventional therapy. While substantial initial responses have been reported, the emergence of resistance to BRAF-targeting drugs is common, leading to reactivation of ERK through different mechanisms and, ultimately, tumor relapse [11]. MEK inhibitors have also been explored as possible therapeutic options in preclinical studies, and were found to restore iodide uptake sufficiently to allow radioiodide treatment, particularly in RAS-mutant-driven thyroid carcinomas [13]. Beyond drug resistance, the high associated toxicity of some systemic drugs limits the dose that can be administered and impacts clinical success [14]. The search for alternative therapies is, therefore, a priority.

ERK forms dimers upon phosphorylation [15], but the physiological implications of ERK dimerization remain obscure. Several studies have used ERK dimerization mutants to address a possible role for ERK dimerization in the nucleo-cytoplasmic shuttling of the protein, with disparate results, from a complete blockade [15] to only delayed [16] ERK nuclear entry. The results of other studies exploring the use of pharmacological inhibitors of ERK dimerization suggest a role for ERK dimers in driving the activation of nuclear or cytoplasmic effectors of the pathway [17]. Recently, Herrero and colleagues identified a water-soluble 3-arylidene-2-oxindole derivative, named DEL-22379, which prevented ERK dimerization with no effect on its phosphorylation [18]. Under conditions of dimerization inhibition, ERK retained the ability to promote activation of nuclear but not cytoplasmic effectors, and DEL-22379 prevented tumor growth in a colorectal patient-derived xenograft mouse model. Importantly, the toxicity associated with the drug was negligible, and the treated cells showed no sign of drug resistance mechanisms common with other inhibitors of this pathway [18]. The Herrero study illustrates two appealing concepts in the search for alternative therapeutic approaches: (i) targeting regulatory protein–protein interactions is a viable option; and (ii) nuclear and cytoplasmic ERK-dependent signaling can be independently targeted.

Given the critical importance of the RAS-ERK pathway for thyroid cancer onset and progression, we aimed to study ERK dimerization dynamics and to analyze the therapeutic effects of DEL-22379 on thyroid carcinogenesis in vitro and in vivo.

## Materials and methods

### Cell culture

The cell lines 8505c and CAL62 were obtained from the Leibniz-Institut DSMZ-German Collection of Microorganism and Cell Cultures, HTH83 cells were donated by Dr. N. E. Heldin (University of Uppsala, Sweden), and the OCUT2 cell line was obtained from Dr. J. Fagin (Memorial Sloan-Kettering Cancer Center, New York). Also, MDCK-hNIS cells were donated by Dr. N. Carrasco (Vanderbilt University, Nashville), and HEK293T and A375P were from Dr. Piero Crespo’s laboratory collection. Cells were authenticated every 6 months by short tandem repeat profiling at the Genomic Facility, Instituto de Investigaciones Biomédicas (Madrid, Spain). Cell lines were used up to passage 10 and were routinely tested for *Mycoplasma* contamination. All cells were grown in Dulbecco's Modified Eagle’s Medium (DMEM) with 10% fetal bovine serum (FBS) (both from ThermoFisher Scientific, Rockford, IL), which was replenished every 2–3 days unless stated otherwise. Epidermal growth factor (EGF; PeproTech, Rocky Hill, NJ) was used at concentrations ranging from 0.1 to 100 ng/ml. The MAPK/MEK inhibitor U0126 (LC Laboratories, Woburn, MA) was dissolved in DMSO and used at a final concentration of 10 µM. DEL-22379 was purchased from Vichem Chemie Research Ltd. (Budapest, Hungary) and was dissolved in DMSO and used at concentrations ranging from 1 to 10 µM.

### Western blotting

Protein extracts were collected in lysis buffer containing 1% NP-40 for native conditions and in RIPA buffer for reducing conditions. Protein concentration was calculated using Bradford’s method [19]. ERK dimerization was evaluated by native-polyacrylamide gel electrophoresis (PAGE), as described [20], whereas SDS-PAGE was used to separate proteins under reducing conditions. In all cases, gels were transferred onto PVDF membranes and blocked with 5% w/v nonfat dry milk in phosphate-buffered saline (PBS) containing 0.1% Tween (blocking buffer). Primary antibodies were diluted in blocking buffer and membranes were incubated at 4 ºC overnight followed by incubation at room temperature for 1 h with the corresponding horseradish peroxidase-conjugated secondary antibody. Immunoreactive bands were visualized with the Enhanced Chemiluminescence Western Blotting Substrate (ThermoFisher Scientific) and detected by autoradiography.

The following primary antibodies were purchased from Santa Cruz Biotechnology (Dallas, TX): ERK2, p^S218/S222^MEK1/2, FOS, JUN, fibronectin, cadherin-2, vimentin, catenin-β1, vinculin, and HA-Tag. The antibodies p^S380^RSK and pp^Thr202/Tyr204^ERK1/2 were purchased from Cell Signaling Technology (Danvers, MA).

### Plasmids and transfections

Cells were transfected with 1 µg HA-ERK2^WT^, HA-ERK2^HL^ or pCEFL (backbone vector) using Lipofectamine 2000 (Invitrogen, Waltham, MA). Twenty-four-hour medium was replaced by a starvation medium (0.2% FBS) for an overnight period, and cells were then pre-treated with 10 µM DEL-22379 or 10 µM U0126 for 30 min, followed by stimulation with 100 ng/ml EGF for 5 min. Plasmids have been described previously [21].

### Reverse transcription-polymerase chain reaction

Total RNA was isolated by TRIzol® (Invitrogen). Equal amounts of RNA were reverse transcribed using a reverse-transcriptase reaction mix (M-MuLV, Promega, Madison, WI). Real-time PCR was performed on the Mx3000 platform (Agilent Technologies Inc., Santa Clara, CA) using the KAPA SYBR FAST qPCR Master Mix (Sigma-Aldrich, St. Louis, MO). mRNA levels were calculated by the 2^–ΔΔCt^ method. Primer sequences are included in Table S1 as Additional File 1.

### Cell viability

Cell viability was measured by live-cell reduction of a tetrazolium dye using the XTT Cell Proliferation Assay Kit (Canvax Biotech, Córdoba, Spain). Cells were seeded into 96-well plates (5000–7500 cells per well) in DMEM/10% FBS. The next day some wells were processed and considered as time 0. After this, the culture medium was replenished every 24 h with the same medium containing 0.1% DMSO (vehicle control) or DEL-22379 (1 µM). Cell plates were processed every 24 h to measure cell viability, which was quantified using a spectrophotometer at a wavelength of 450 nm. Results are expressed as the relative fold-change of the mean (standard deviation [SD]) of three independent experiments performed in sextuplicate.

### 5-bromo-2′-deoxyuridine incorporation assay

Cells were seeded and grown as explained above for the cell viability analysis. The culture medium was replenished every 24 h with the same medium containing 0.1% DMSO (vehicle control), 1 µM DEL-22379 (D1), 5 µM DEL-22379 (D5), or 10 µM U0126. Cells were collected after 72 h for 5-bromo-2′-deoxyuridine (BrdU) incorporation measurements using the Cell Proliferation ELISA, BrdU (chemiluminescent) kit from Roche (Basel, Switzerland). Results are expressed as the relative fold-change of the mean (SD) of three independent experiments performed in quintuplicate.

### Apoptosis

Cells were classified as viable, dead or early apoptotic using a membrane permeability/dead cell apoptosis kit with YO-PRO-1® and propidium iodide for flow cytometry (Invitrogen). In total, 1.5 × 10^6^ cells were seeded into 10-cm cell plates in DMEM with 10% FBS. The next day, the culture medium was replenished with the same medium containing 0.1% DMSO (vehicle control), DEL-22379 (1 or 10 µM) or U0126 (10 µM). After 24 h, cells were recovered with trypsin and processed for flow cytometry analysis. Results are expressed as the percentage of the mean (SD) of the number of viable, apoptotic and dead cells from 3 independent experiments.

### Immunofluorescence

A total of 2 × 10^5^ cells were seeded into each well of a 6-cm plate containing coverslips, in DMEM with 10% FBS. On the next day, the culture medium was replenished every 24 h with the same medium containing 0.1% DMSO (vehicle control), DEL-22379 (1 µM) or U0126 (10 µM). After 48 h, coverslips were fixed in 4% paraformaldehyde (PFA), blocked in 0.1% Tween/PBS with 3% bovine serum albumin (BSA) for 1 h, incubated with primary antibodies for 1 h, washed 3 times in 0.1% Tween/PBS, and then incubated with fluorophore-conjugated secondary antibodies and rhodamine-conjugated phalloidin for 1 h. Coverslips were washed 3 times in 0.1% Tween/PBS, with the last wash containing 1:5000 4′,6-diamidino-2-phenylindole (DAPI) for nuclear staining. Coverslips were then mounted on ProLong™ Diamond Antifade Mountant (Invitrogen) and analyzed with an LSM710 confocal microscope (Carl-Zeiss AG, Oberkochen, Germany) using a 63 × objective. Images were analyzed with ImageJ software (NIH, Bethesda, MD). As intensity was not considered in this analysis, brightness and contrast were adjusted to provide clear visualization. A 20-µm scale bar is included in all images. A representative image from at least 3 independent experiments is shown.

### Adhesion

Cells were seeded into 96-well plates pre-coated with Geltrex™ (Thermo Fisher Scientific), a reduced growth factor basement membrane matrix. Coated wells were first washed twice with DMEM containing 0.1% BSA (washing buffer) and then blocked with DMEM containing 0.5% BSA (blocking buffer) for 1 h at 37ºC in a cell incubator. Plates were finally washed with washing buffer. Trypsinized cells were incubated in suspension with DMEM and 10% FBS containing 0.1% DMSO (vehicle control), DEL-22379 (1, 5 or 10 µM) or U0126 (10 µM). Then, 5 × 10^3^ (8505c, OCUT2 and HTH83) or 1 × 10^4^ (CAL62) cells per well were seeded into plates and allowed to adhere for 30 and 45 min or for 20 and 45 min, respectively. Plates were washed twice with cold PBS, fixed for 10 min with 4% PFA, washed and stained with a 2% ethanol solution of 5 mg/ml crystal violet for 10 min at room temperature, washed thoroughly with distilled water, and allowed to air-dry completely. Finally, 2% SDS was added for 30 min at room temperature and the optical density (500 nm) was quantified in a spectrophotometer. Three independent experiments in quintuplicate were performed. Results are expressed as mean (SD) relative to control (DMSO).

### Matrigel-invasion assay

The invasion capacity of cells was determined using Corning® BioCoat™ Matrigel® Invasion Chambers (Corning, New York, NY). Cells were seeded at sub-confluence in 6-well plates in DMEM with 10% FBS. The next day, the culture medium was replaced with DMEM containing 0.2% BSA for 24 h. Subsequently, DMSO (vehicle control), DEL-22379 (1 or 2.5 µM) or U0126 (10 µM) were added for 30 min. Cells were recovered with trypsin and 5 × 10^4^ (8505c, CAL62, HTH83) or 2.5 U0126 10^4^ (OCUT2) cells were seeded in DMEM with 0.2% BSA, containing the inhibitors or vehicle control, into the upper chamber of 24-well plates included in the kit. Cells were allowed to invade through an 8-µm pore-size PET membrane (covered with a thin Matrigel® layer) for 24 h, using 20% FBS in DMEM as a chemoattractant in the lower chamber of the wells. Non-invasive cells were removed, membranes were fixed in 4% PFA, stained with a crystal violet solution, and photographed under a phase-contrast microscope at 20 × magnification. The number of invasive cells was estimated using ImageJ. Data represent the relative mean (SD) of the number of invasive cells, obtained from 4-fields per chamber, from 3 independent experiments performed in duplicate.

### Time lapse-wound healing assay

Cells were seeded into 12-well plates at 90% confluence in DMEM with 10% FBS. After an overnight period in DMEM with 0.2% BSA (100% confluence), mitomycin C (10 µg/ml; Sigma-Aldrich) was added for 2 h to halt cell proliferation. Subsequently, the cell monolayer was scratched to create a cell-free gap of consistent width, and plates were washed twice with PBS to eliminate cellular remnants. Cells were then cultured in DMEM with 10% FBS containing DMSO (vehicle control), DEL-22379 (1 or 2.5 µM) or U0126 (10 µM), and wound closure was monitored for 24 h in a live-cell microscope (Cell Observer, Carl-Zeiss AG). Wound areas were quantified at times 0 and 20 h using ImageJ and migration rates were estimated by subtracting the cell-free area from time-20 h to time-0. The migration rate relative to the vehicle control was expressed as the mean (SD) of three independent experiments performed in duplicate.

### Stranded mRNA library preparation and sequencing

RNA was extracted using the PureLink™ RNA Mini Kit (ThermoFisher Scientific) from 8505c or CAL62 cells grown for 24 h in the presence of 10% FBS plus DMSO (vehicle), DEL-22379 (1 or 10 µM), or U0126 (10 µM) in two biological replicates. Total RNA was assayed for quantity and quality using the Qubit RNA BR Assay Kit on the Qubit 2.0 Fluorometer (Life Technologies, Carlsbad, CA) and the RNA 6000 Nano Assay on a Bioanalyzer 2100 (Agilent). RNA-Seq libraries were prepared using the TruSeq Stranded mRNA LT Sample Prep Kit protocol (Illumina Inc., San Diego, CA). Libraries were sequenced on a NovaSeq 6000 platform (Illumina) with a read length of 2 × 51 bp following the manufacturer’s protocol for dual indexing. Image analysis, base calling and quality scoring of the runs were processed using the manufacturer’s software Real-Time Analysis (RTA v3.4.4) and was followed by the generation of FASTQ sequence files.

Gene enrichment analysis was performed with g:Profiler (https://biit.cs.ut.ee/gprofiler/gost) using GO molecular function (MF), cellular component (CC) and biological process (BP) as data sources.

### Immunohistochemistry

Mouse thyroid tumor biopsies were fixed in 4% PFA for 16 h, embedded in paraffin and cut into 3-µm sections. Tissue slides were deparaffinized and heated to expose antigens using the Real Target Retrieval Solution containing pH 6.0 citrate buffer (Dako, Glostrup, Denmark). Samples were immunostained using an antibody to Ki67 (Abcam, Cambridge, UK) or to pERK1/2 (Invitrogen) with the REAL™ EnVision™ Detection System, Peroxidase/DAB + (Dako) and then counterstained with hematoxylin.

The nuclear Ki67-positive cell ratio was expressed in a violin plot as the median and quartiles of 3–5 fields per sample (8505c: Control group, *N* = 8; DEL-22379 group, *N* = 9. CAL62: Control group, *N* = 14; DEL-22379 group, *N* = 11), at 20 × using ImageJ.

For evaluation of pERK1/2 protein levels, a histoscore (H-score) was calculated by semi-quantitative assessment of the intensity and percentage of positive cells. Staining intensity was graded as follows: 0, no staining; 1, weak; 2, moderate; and strong staining, 3. The percentage of positive cells was divided into four grades: 1, 0–25% staining; 2, 26–50% staining; 3, 51–75% staining; and 4, 76–100% staining. The H-score was expressed as the mean (SD) of 3–5 fields at 20 × magnification.

### In vivo tumor progression and tumor size

The CMV-Firefly Luc-IRES-EGFP vector was provided by Dr J. Blanco (IQAC-CSIC, Barcelona, Spain), and CAL62 cells stably expressing the vector (CAL62-LUC) were generated by Dr E. Mato (IIB-UAB, Sant Pau, Spain).

The vector encoding LUC-mCherry (pCDH-EF1a-eFFly-mCherry; Addgene plasmid #104833) was a kind gift from Dr A. Cano (UAM, Madrid, Spain). Lentivirus production and cell infection were performed as described [22]. At 48 h following infection, 8505c cells expressing LUC-mCherry were sorted using a FACSVantage SE Flow Cytometer (Becton Dickinson) at the Universidad Autónoma de Madrid.

To analyze the effects of DEL-22379 on in vivo tumor progression, we used an orthotopic mouse model of ATC. A total of 5 × 10^4^ 8505c or CAL62 cells, expressing luciferase, were injected into the right lobe of the thyroid of immunocompromised nude mice. Tumor formation was monitored weekly by measuring luciferase activity for 30 s in an IVIS® Lumina II In Vivo Imaging System (Perkin Elmer, Walthan, MA) 10 min after subcutaneous injection of 4 mg d-luciferin (Promega).

Primary tumors could be clearly detected 1 week after cell inoculation and the luminescent signal elicited was considered as time 0. Tumor engraftment achievement was 80% for 8505c cells and 93% for CAL62 cells. Animals were separated into 2 groups with similar luminescent signals and mice were treated with vehicle or DEL-22379 (15 mg/kg) by intraperitoneal injection every 12 h. During the course of the experiment, two mice from the 8505c/DEL-22379 group were excluded because of the incorrect location of the tumor in one case and chronic priapism (leading to euthanasia) in the other case. The signal elicited by tumor cells was analyzed weekly using in vivo imaging, and is expressed as the relative mean (standard error of mean, SEM) of the radiance (p/sec/cm^2^/sr) fold-change: 8505c: Control group, *N* = 12; DEL-22379 group, *N* = 10. CAL62: Control group, *N* = 14; DEL-22379 group, *N* = 14.

Final tumor volume was determined ex vivo by measuring height (*H*) and width (*W*) with a caliper, and applying the formula *V* = (*H* × *W* × *W*)/2. Results are depicted in a violin plot, showing median and quartiles.

The presence of lung metastases was determined ex vivo by subcutaneous injection of 4 mg of d-luciferin 10 min before euthanasia: (8505c: Control group, *N* = 12; DEL-22379 group, *N* = 10. CAL62: Control group, *N* = 14; DEL-22379 group, *N* = 13).

### Statistical analysis

Two-way ANOVA was used in cell viability and in vivo tumor growth assays; and a two-tailed Student’s paired *t* test or unpaired *t* test was used, respectively, for cell lines and mouse-group comparisons. Differences were considered significant at (*)*p* = 0.05–0.01, (**)*p* = 0.01–0.001 and (***)*p* < 0.001.

## Results

### Impairment of ERK dimerization by DEL-22379 in thyroid tumor cells is dependent on the oncogenic driver

The 3-arylidene-2-oxindole derivative DEL-22379 has been previously described as an inhibitor of ERK dimerization, with no effect on ERK phosphorylation [18]. To examine the potential effects of the inhibitor on human ATC-derived cell lines on the basis of the initial driver mutation, we chose two cells carrying the BRAF^V600E^ mutation (8505c and OCUT2) and two cell lines with K-RAS^G12R^ or H-RAS^Q61R^ mutations (CAL62 and HTH83, respectively).

We first monitored ERK dimer formation in cells treated with DEL-22379 or with the MEK inhibitor U0126, using the same concentrations and exposure times as previously described [18]. Cells were deprived of serum and growth factors overnight before being treated for 30 min with 10 µM DEL-22379, 10 µM U0126 or vehicle (DMSO) and then subsequently stimulated with 100 ng/ml EGF for 5 min. ERK dimer formation was analyzed by native gel electrophoresis, which separates ERK dimers from monomers based on migration, as described [18].

**Fig. 1:**
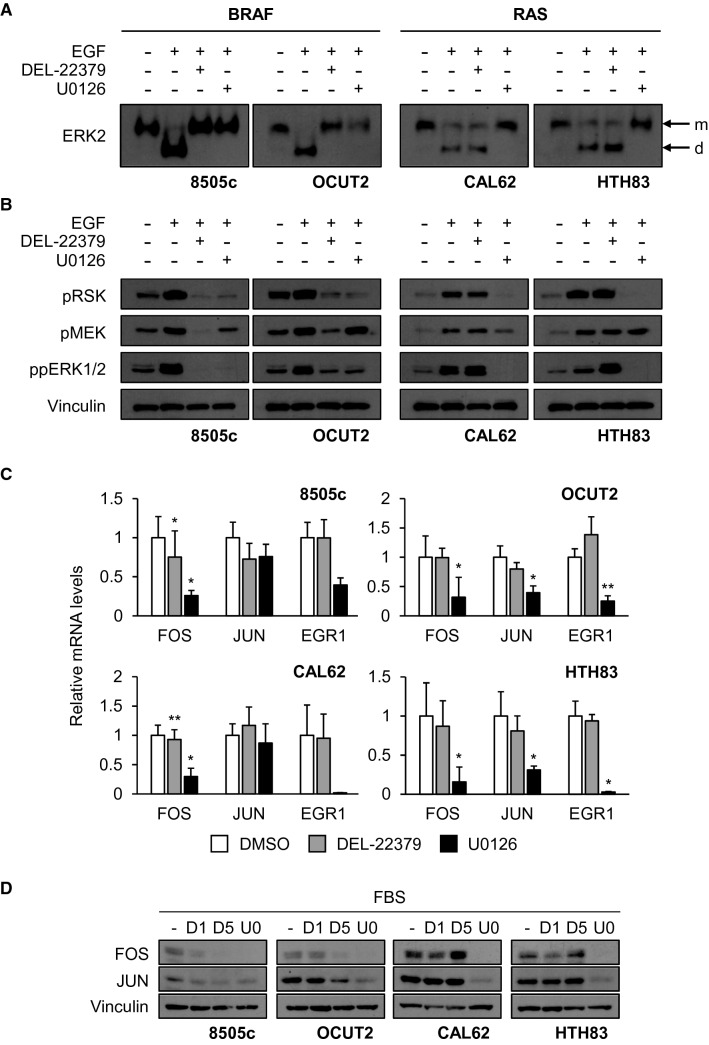
Impact of DEL-22379 and U0126 on ERK dimerization and signaling in thyroid tumor BRAF- and RAS-mutant cells. 8505c and OCUT2 cells carry BRAF^V600E^ mutations and CAL62 and HTH83 cells carry K-, and H-RAS mutations, respectively. Western blot assays are representative from 3 independent experiments. **A** Native western blot assay of cells pre-treated for 30 min with 10 µM DEL-22379, 10 µM U0126 or vehicle before stimulation for 5 min with 100 ng/ml EGF. For ERK2 protein analysis, the upper band in the gels corresponds to the monomeric (m) form and the lower band to the dimeric (d) form. **B** Protein extracts from (**A**) were separated in a SDS-PAGE western blot assay. p^T202/Y204^ERK1/2 was detected to monitor ERK activation, p^S380^RSK was detected to monitor ERK cytoplasmic activity, p^S218/S222^MEK1/2 was detected to show upstream activation of the pathway and vinculin was used as a loading control. **C** mRNA levels of the ERK nuclear effectors *FOS*, *JUN* and *EGR1* after 48 h in the presence of 10% FBS plus 1 µM DEL-22379, 10 µM U0126 or vehicle (DMSO), estimated by RT-qPCR. Results are expressed as mean (SD) of at least 3 independent experiments. Statistical significance of differences elicited by treatments compared with a vehicle was calculated using a two-tailed t-test: (*)*p* = 0.05–0.01, (**)*p* = 0.01–0.001. **D** Representative western blot of three independent experiments showing FOS and JUN protein levels after 48 h in the presence of 10% FBS plus DMSO (−), 1 µM DEL-22379 (D1), 5 µM DEL-22379 (D5) or 10 µM U0126 (U0). Vinculin is shown as a loading control

We detected ERK dimerization in BRAF-mutant cells in response to EGF, and this was prevented by pre-treatment of cells with DEL-22379 or U0126 (Fig. 1A, left panels). By contrast, pre-treatment with DEL-22379 but not U0126 failed to impair ERK dimerization in EGF-treated RAS-mutant cells (Fig. 1A, right panels). The lack of effect of DEL-22379 on EGF-treated RAS-mutant cells was not due to the concentration of EGF used to activate ERK, as an EGF dose closer to physiological levels (10 ng/ml) resulted in a high degree of ERK dimerization in both 8505c and CAL62 cell lines (Additional File 2A). The dose of the inhibitor required to impair ERK dimerization in 8505c cells was substantially lower at these lower EGF doses; nevertheless, CAL62 cells remained insensitive to the inhibitor at all doses tested (Additional File 2B).

Overall our data suggest that, in contrast to what has been reported in other tumor types [18], DEL-22379 is functional only in thyroid tumor cells harboring BRAF mutations.

### Impact of DEL-22379 on ERK phosphorylation and activation of downstream effectors is dependent on the oncogenic driver in thyroid tumor cells

ERK phosphorylation is a pre-requisite for its dimerization and, accordingly, inhibition of MEK activity prevents ERK dimerization. DEL-22379 was previously described to have no effect on ERK phosphorylation [18]. Because dimerization is needed to connect ERK scaffolds to cognate cytoplasmic substrates [21], DEL-22379 impairs the activation of ERK cytoplasmic effectors, such as RSK, but not that of nuclear effectors, which associate with ERK monomers. To test this mechanism in thyroid tumor cells, we repeated the preceding study and examined ERK phosphorylation using western blotting.

Unexpectedly, DEL-22379 impaired both ERK and RSK phosphorylation in response to short-term EGF treatment in BRAF-mutant cells, but not in RAS-mutant cells (Fig. 1B). In addition, phosphorylation of MEK, the upstream activator of ERK, was blocked by the inhibitor to the same extent as that observed for ERK (Fig. 1B).

These results parallel the findings of the drug on ERK dimerization, overall indicating that the evident reduction or enhancement of ERK dimerization is linked to the loss or gain of ERK phosphorylation, and demonstrating that the inhibitory effect of DEL-22379 in BRAF-mutant thyroid cells occurs upstream in the pathway.

To further clarify whether DEL-22379 acts on ERK phosphorylation or dimerization, 8505c cells were transfected with wild-type ERK2 (ERK2^WT^) or an ERK2-dimerization-deficient mutant (ERK2 H176E, L_4_A; ERK2^HL^ hereafter), previously described [15], which retains phosphorylation by MEK. DEL-22379 treatment impaired ERK2 phosphorylation under every condition tested, unequivocally proving that DEL-22379 actions on ERK dimerization are due to the loss of ERK phosphorylation (Additional File 3A).

These results differ from previous observations [18], and might be explained by the different cellular system used in the present study. To clarify this, we analyzed the effect of DEL-22379 and U0126 on HEK293T and A375P cells, which were previously used to characterize the effects of the drug on ERK dimerization and phosphorylation [18]. DEL-22379 treatment impaired ERK dimerization, with no effects on pERK levels, which were effectively suppressed by MEK inhibition (Additional File 3B).

ERK activates and promotes the expression of several early-response transcription factors in the nucleus, such as FOS, JUN and EGR1 [23]. To analyze the effects of DEL-22379 on ERK-nuclear effectors, we treated cells growing in the presence of 10% FBS with 1 µM DEL-22379, the maximal dose tolerated by mice, or the MEK inhibitor U0126 (10 µM) for 48 h and mRNA levels of the response genes were quantified by RT-qPCR. Results showed that *FOS*, *JUN* and *EGR1* expression decreased to different extents in most of the cell lines upon MEK inhibition (Fig. 1C); however, gene expression was scarcely affected by 1 µM DEL-22379.

To confirm these results at the protein level, we performed western blotting of cell extracts after treatment with increasing concentrations of DEL-22379 or with U0126 (10 µM) for 48 h. Consistent with the gene expression analysis, steady-state levels of FOS and JUN were markedly lower in all U0126-treated cell lines, and were dose-dependently reduced upon DEL-22379 treatment only in BRAF-mutant cells (Fig. 1D). The lower expression of ERK nuclear effectors correlates with the short-term loss of ERK phosphorylation elicited by DEL-22379 pre-treatment, suggesting that this response to the drug is due to ERK inactivation.

### DEL-22379 has minimal effects on thyroid tumor cell viability and apoptosis

Our findings in thyroid tumor cells are different from those reported in melanoma, hepatocellular carcinoma (HCC) and colorectal carcinoma (CRC) cells [18], indicating that the actions of the inhibitor on ERK dimerization likely depend on the cell type.

**Fig. 2:**
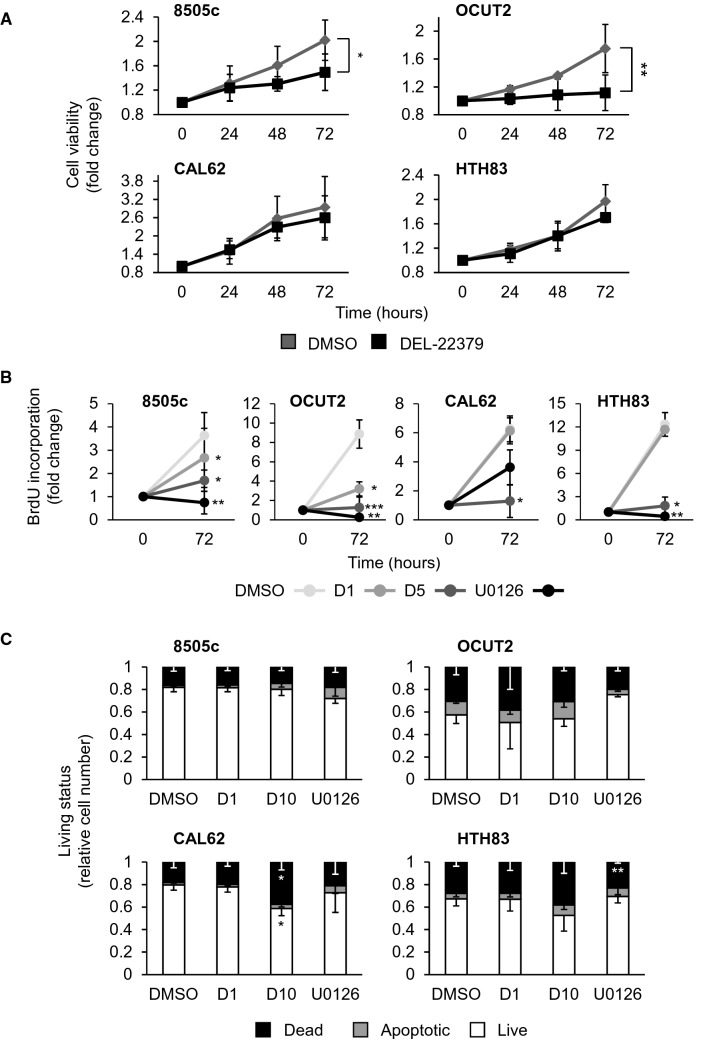
Impact of DEL-22379 on cell viability and apoptosis in thyroid tumor cells. 8505c and OCUT2 cells carry BRAF^V600E^ and CAL62 and HTH83 cells carry K-, and H-RAS mutations, respectively. **A** XTT-reduction cell viability assay showing fold-change of living cells over time: 24, 48 and 72 h (h), in the presence of 10% FBS plus 1 µM DEL-22379 or vehicle (DMSO). Results are expressed as mean (SD) of at least 3 independent experiments. Statistical significance of differences elicited by DEL-22379 was calculated by two-way ANOVA (*)*p* = 0.05–0.01, (**)*p* = 0.01–0.001. **B** BrdU incorporation assay after 72 h of treatment with 1 µM (D1) or 5 µM (D5) DEL-22379, 10 µM U0126 or vehicle (DMSO). Results are expressed as mean (SD) of fold-change and statistical significance of differences compared with a vehicle was calculated by paired *t* test. (*) *p* = 0.05–0.01, (**)*p* = 0.01–0.001, (***) *p* < 0.001. **C** YOPRO-1/PI incorporation assay showing percentage of live, apoptotic and necrotic cells after 24 h in the presence of 10% FBS plus vehicle (DMSO), 1 µM DEL-22379 (D1), 10 µM DEL-22379 (D10) or 10 µM U0126. Results are expressed as the percentage of mean (SD) of cell number. Statistical significance of differences elicited by the treatments compared with a vehicle was calculated using a two-tailed *t* test. (*)*p* = 0.05–0.01, (**)*p* = 0.01–0.001

We next assessed the effects of DEL-22379 on cell viability using an XTT-based reduction assay. BRAF-mutant cell lines stimulated with 10% FBS and pre-treated with 1 µM DEL-22379 showed a significant decrease in cell viability over time, while no significant effect was found for RAS-mutant cell lines (Fig. 2A). A BrdU incorporation assay confirmed this observation. Used at a concentration of 1 µM, DEL-22379 decreased the proliferation only in BRAF-mutant cells, while at a 5 µM concentration growth inhibition was clear in all cell lines (Fig. 2B).

DEL-22379 was previously shown to trigger apoptosis in melanoma- and CRC-derived cell lines depending on their mutational status, and the cytotoxic effects were more pronounced in BRAF-mutant cells, variable in RAS-mutant cells and negligible in cells wild-type for both oncogenes [18]. To assess the apoptotic action of DEL-22379 on ATC-derived tumor cells, we used flow cytometry to measure plasma membrane integrity by YoPro/PI staining. No significant changes were found in the number of dead and apoptotic BRAF- or RAS-mutant cells in the presence of 1 µM DEL-22379, and a higher concentration of inhibitor (10 µM) decreased cell viability only in RAS-mutant cells (*p* = 0.0626 in HTH83 cells) (Fig. 2C). By contrast, MEK inhibition with U0126 failed to trigger apoptosis in any of the cell lines tested (Fig. 2C).

These results indicate that low doses of DEL-22379 are likely cytostatic in BRAF-mutant cells, and a higher dose is required to promote cytotoxic effects, but only in RAS-mutant cells.

### Impact of DEL-22379 on metastasis-related cellular processes in thyroid cancer cells

Because ATC is associated with a high incidence of local and distant spreading, leading to a rapidly progressive clinical course and dismal outcome [24], we next analyzed the impact of DEL-22379 on cellular processes required for metastatic dissemination.

#### Cytoskeleton and cell adhesion

**Fig. 3:**
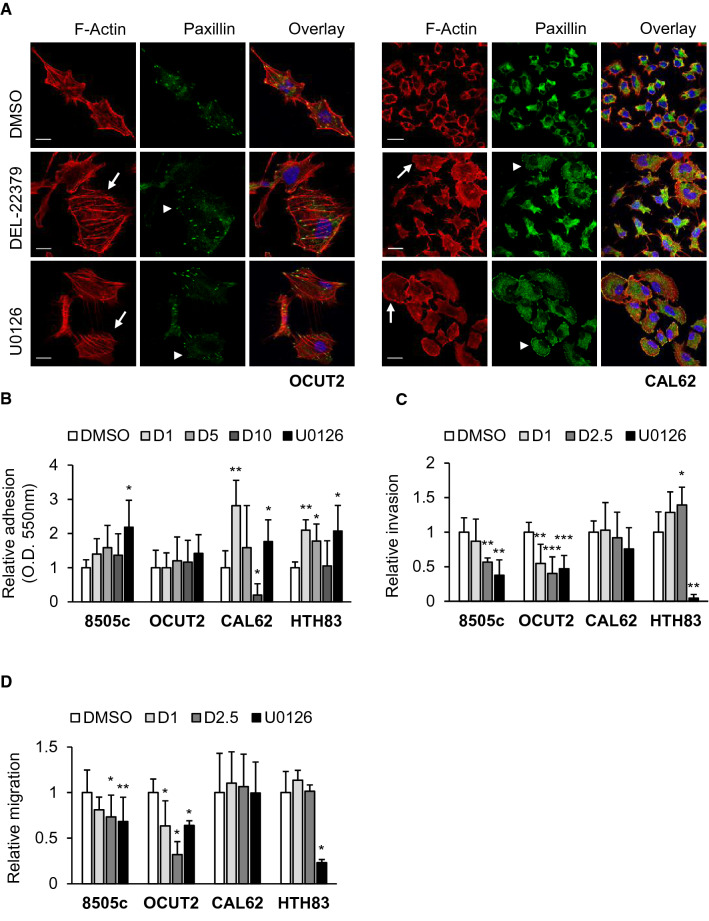
Effects of DEL-22379 on cell spreading, adhesion, migration and invasion of thyroid-tumor cells. 8505c and OCUT2 cells carry BRAF^V600E^ and CAL62 and HTH83 cells carry K-, and H-RAS mutations, respectively. **A** Confocal immunofluorescence showing F-actin (red), paxillin (green) and overlay plus DAPI (blue) nuclear staining in OCUT2 (left panels) and CAL62 (right panels) cells, in the presence of 10% FBS plus vehicle (DMSO), 1 µM DEL-22379 or 10 µM U0126 for 48 h: scale bar is included in the red images, 20 µm. Arrows point to cells with increased spreading and arrowheads to focal adhesion sites. **B** Cell adhesion assay showing the relative mean (SD) from at least 3 independent experiments of the number of adherent cells 30 (8505c), 45 (OCUT2 and CAL62) and 20 (HTH83) min after seeding in 10% FBS-medium containing vehicle (DMSO), 1 µM DEL-22379 (D1), 5 µM DEL-22379 (D5), 10 µM DEL-22379 (D10) or 10 µM U0126. Cells were pre-treated for 30 min with the different agents in suspension before seeding. **C** Matrigel invasion assay showing the relative mean (SD) from 3 independent experiments of the number of invasive cells, after 24 h in serum-free medium containing vehicle (DMSO), 1 µM DEL-22379 (D1), 2.5 µM DEL-22379 (D2.5) or 10 µM U0126. **D** Wound healing assay showing the relative mean (SD) from 3 independent experiments of occupied cell-free area after 20 h in the presence of 10% FBS plus vehicle (DMSO), 1 µM DEL-22379 (D1), 2.5 µM DEL-22379 (D2.5) or 10 µM U0126. **B**–**D** Statistical significance of differences elicited by the treatments compared with a vehicle was estimated using a two-tailed t-test. (*)*p* = 0.05–0.01, (**)*p* = 0.01–0.001, (***)*p* < 0.001

Actin cytoskeleton and focal adhesions (FAs) are important in the development and progression of metastases, and are involved in adhesion, migration and invasion. As the ERK pathway is a known regulator of cytoskeleton dynamics [25], we examined the effects of DEL-22379 on both structures by immunofluorescence using phalloidin to visualize F-actin and paxillin as a marker of FAs. Results showed that treatment of cells with 1 µM DEL-22379 or 10 µM U0126 for 48 h triggered different alterations to the actin cytoskeleton (Fig. 3A). Under normal culture conditions, OCUT2 cells have an elongated morphology and CAL62 cells are small and round. The addition of either compound increased spreading in both cell types, and this was accompanied by the formation of actin stress fibers with loss of directionality and disorganization of FAs specifically in OCUT2 cells. FAs were not detected in CAL62 cells growing under normal culture conditions; however, DEL-22379, and to a greater extent U0126, triggered the formation of these structures (Fig. 3A).

As our results suggest that DEL-22379 and U0126 increase cell adhesiveness, we next performed adhesion assays on a growth factor-reduced basement membrane matrix. While the cytoskeletal changes triggered by DEL-22379 were observed after 48 h, the ability of some cell types to adhere to the matrix substrate occurred quickly after treatment (Fig. 3B). Specifically, treatment of BRAF-mutant cells with increasing concentrations of DEL-22379 for 30 min did not affect adhesion, but RAS-mutant cells showed a marked increase in adhesion with 1 or 5 µM DEL-22379. This effect was lost at a higher concentration of the inhibitor (10 µM), especially in CAL62 cells where adhesion was almost totally inhibited. Notably, MEK inhibition with U0126 increased the number of adherent cells in most of the cell lines, confirming the immunofluorescence findings. Altogether, these observations indicate that the actions of DEL-22379 on cell adhesion occur very quickly and are unlikely to be related to ERK activation status, pointing to off-target effects of the drug, especially on RAS-mutant cells.

#### Cellular invasion and migration

Given the above findings, we next tested the action of DEL-22379 on cell invasion using a Matrigel™-based invasion chamber assay (Fig. 3C). Results showed that DEL-22379 dose-dependently decreased the invasion ability of BRAF-mutant cells. By contrast, DEL-22379 slightly increased the invasion capacity of HTH83 cells, while no significant effect was observed on CAL62 cells. MEK inhibition with U0126 reduced the number of invasive cells in all cases, with the only exception being CAL62 cells, which showed a striking ERK-independence in terms of invasion.

We next performed scratch wound-healing assays to analyze the effects of DEL-22379 on cell migration, which is fundamental to invasion. Cells were recorded for 20–24 h using an in vivo cell observer. A representative video for each experimental condition is provided as supplementary material (Additional File 4). Results showed that DEL-22379 significantly reduced the motility of BRAF-mutant cells, but had no effect on RAS-mutant cells (Fig. 3D). Of note, the addition of DEL-22379 to 8505c cells, even at a low dose, promoted a similar degree of impaired cell migration to that observed with MEK inhibition, whereas 2.5 µM DEL-22379 was even more effective than U0126 in OCUT2 cells. As the MEK inhibitor is highly efficient in preventing ERK phosphorylation (Additional File 5), these results suggest that ERK inhibition is not the only mechanism responsible for the action of DEL-22379 on cell motility, likely reflecting off-target effects of the molecule.

#### Mesenchymal state

The acquisition of mesenchymal features is fundamental for metastatic cells to establish secondary tumors [26]. To analyze how the mesenchymal phenotype is affected by DEL-22379 or U0126, we used RT-qPCR to measure the expression of instructive mesenchymal transcription factors and structural genes in cells stimulated with 10% FBS for 48 h.

**Fig. 4:**
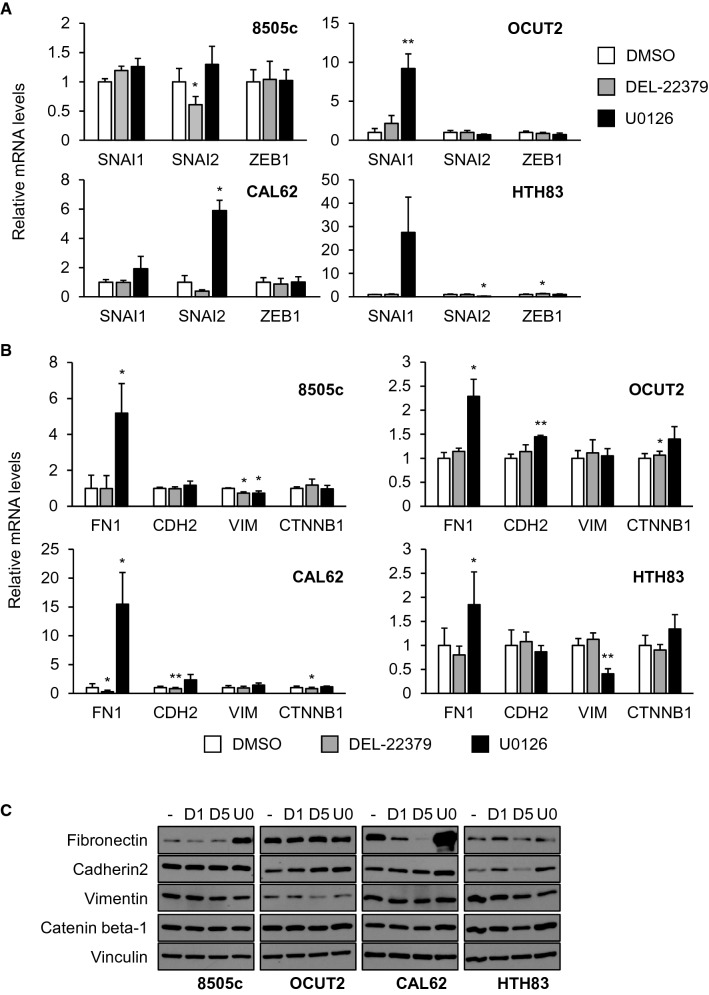
Regulation of thyroid tumor cell mesenchymal state by DEL-22379. 8505c and OCUT2 cells carry BRAF^V600E^ and CAL62 and HTH83 cells carry K-, and H-RAS mutations, respectively. mRNA levels of mesenchymal transcription factors (**A**), *SNAI1*, *SNAI2* and *ZEB1*, and mesenchymal markers (**B**), *FN1*, *CDH2*, *VIM* and *CCTNB1*, after 48 h in the presence of 10% FBS plus 1 µM DEL-22379, 10 µM U0126 or vehicle (DMSO), measured by RT-qPCR. Results are expressed as mean (SD) of at least 3 independent experiments. Statistical significance of differences elicited by treatments compared with a vehicle was calculated using a two-tailed *t* test: (*)*p* = 0.05–0.01, (**)*p* = 0.01–0.001. **D** Representative western blot assay of three independent experiments showing fibronectin, cadherin2, vimentin and catenin beta-1 protein levels after 48 h in the presence of 10% FBS plus DMSO (−), 1 µM DEL-22379 (D1), 5 µM DEL-22379 (D5) or 10 µM U0126 (U0). Vinculin is shown as a loading control

Results showed that the expression of mesenchymal transcription factors was differentially affected depending on the cell type and treatment. In general terms, DEL-22379 treatment failed to promote major changes in mRNA expression, except for a significant decrease in the expression of *SNAI2* in 8505c cells (Fig. 4A). By contrast, MEK inhibition stimulated a marked increase in the expression of *SNAI1* and/or *SNAI2* in the remaining cell lines (Fig. 4A).

Regarding the structural mesenchymal genes, *FN1* (fibronectin) expression increased to different extents in all cell types upon MEK inhibition, particularly in 8505c and CAL62 cells, which showed a 4- to 15-fold overexpression (Fig. 4B). Notably, DEL-22379 strongly decreased *FN1* levels in CAL62 cells, with no significant changes in other cell types. Minor or non-significant changes were found in the expression of other mesenchymal markers with the exception of a marked decrease in *VIM* (vimentin) expression in HTH83 cells upon MEK inhibition (Fig. 4B).

We repeated the experiment and examined protein levels by western blotting (Fig. 4C). Similar to the gene analysis, steady-state levels of fibronectin increased in 8505c and CAL62 cells upon MEK inhibition, whereas its levels decreased in a dose-dependent manner in CAL62 cells in the presence of DEL-22379. Also, DEL-22379 and U0126 treatment reduced the levels of vimentin in 8505c and HTH83 cells, whereas cadherin2 expression increased in OCUT2 cells growing in the presence of either compound.

These results suggest that DEL-22379 treatment elicits only minor changes in the mesenchymal state of the cells, whereas MEK inhibition promotes a selective increase in the expression of mesenchymal transcription factors and markers.

Overall, these findings suggest that the effects of DEL-22379 are likely not solely related to the inhibition of ERK dimerization and/or phosphorylation, and might include off-target actions, especially at the 10 µM dose. Of note, blocking ERK phosphorylation by inhibiting MEK enhanced the metastatic trails of ATC-derived cell lines, including adhesion and mesenchymal gene expression. This could represent an intrinsic resistance mechanism to the silencing of this crucial kinase cascade.

### Impact of DEL-22379 on the transcriptional landscape of thyroid tumor cells

To further assess the effect of DEL-22379 on thyroid tumor cell transcription, we performed RNA-seq on 8505c and CAL62 cell lines grown for 24 h in the presence of vehicle, DEL-22379 (1 and 10 µM) or U0126 (10 µM). Genes were considered differentially expressed with a *p*-adjusted value (*p*-adj) of < 0.01 and with shrunken log-fold change (shrFC) > 1.5 or < − 1.5. Principal component analyses (Additional File 6A) and sample-to-sample heatmaps (Additional File 6B) of the individual samples revealed that the main variance was between cell lines. Low-dose DEL-22379 treatment elicited minor effects in both cell lines, whereas high-dose DEL-22379 and U0126 treatment of CAL62 cells caused gene expression changes in opposite directions.

Only 8 genes in 8505c cells and 1 gene in CAL62 cells were differentially expressed after treatment with 1 µM DEL-22379. By contrast, the higher dose of DEL-22379 and U0126 affected the expression of a great number of genes (Additional File 6C). Although not meeting the *p-*adj and shrFC criteria, from the 1 µM DEL-22379 groups, the top 50 differentially expressed genes in 8505c and 4 genes in CAL62, with *p*-adj < 0.1 and independently of shrFC, were used to have a glimpse of overlapping genes at this concentration. The complete list of genes used in Additional file 6D–G is shown as Additional File 7. Venn diagrams were used to compare overlapping under- (Additional File 6D) and overexpressed (Additional File 6E) genes between cell lines and treatments. The results indicated that 10 µM DEL-22379 and U0126 regulated the transcriptomic profile of 8505c cells more similarly than in CAL62 cells, supporting the contrasting effects of DEL-22379 on the cell lines.

Gene enrichment analysis showed that the underexpressed genes in 8505c cells (Additional File 6F) in either condition were related to cytokine and inflammatory response, and cell differentiation. Underexpressed genes in cells treated with 10 µM DEL-22379 or U0126 were also involved in MAPK activation and tyrosine kinase-related signaling, and in cellular processes such as proliferation, adhesion, migration, chemotaxis and angiogenesis. Overexpressed genes in U0126-treated cells were related to lipid biosynthesis in any condition, and to extracellular matrix (ECM) organization.

With regard to CAL62 cells (Additional File 6G), the underexpressed genes after 10 µM DEL-22379 treatment and the overexpressed genes after 10 µM U0126 treatment were associated with cell adhesion, cell–cell junction, ECM organization or mesenchymal and stem cell development. Overexpressed genes in DEL-22379-treated cells and underexpressed in U0126-treated cells were associated with cell cycle and DNA processing in both cases, and additionally with response to cytokines, cell differentiation and adhesion in the former, and DNA damage and repair in the latter.

Overall, the transcriptomics analyses support the findings of the in vitro studies, highlighting the opposite effects of DEL-22379 depending on the cell type, and suggesting independent actions of the drug non-related to ERK activation status.

### In vivo tumor progression

**Fig. 5:**
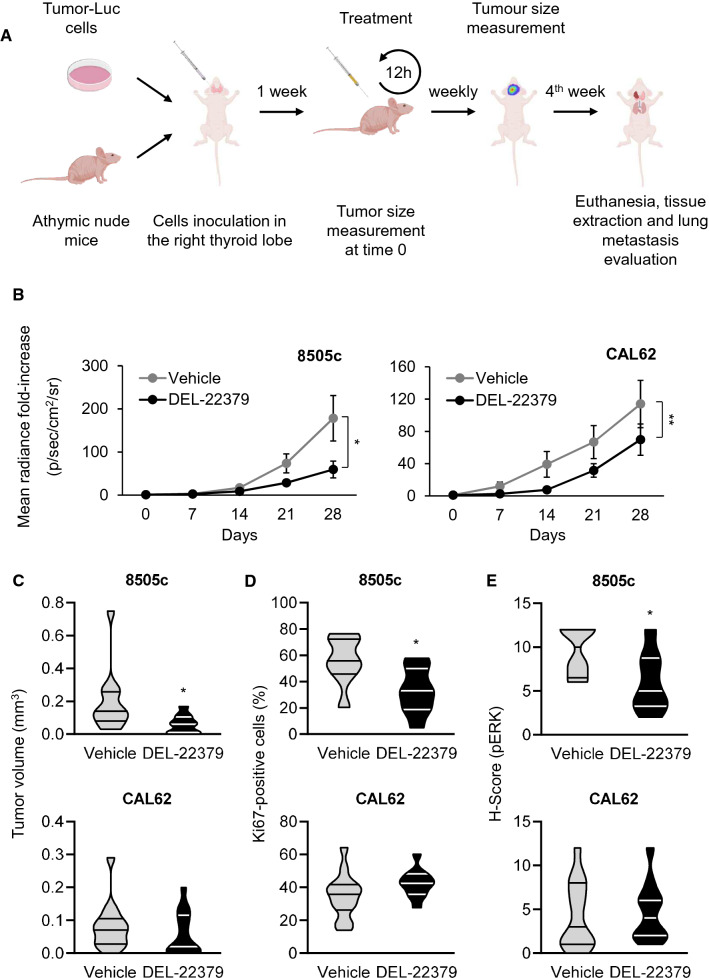
Effects of DEL-22379 on in vivo tumor growth. **A** Graphical explanation of in vivo tumor engraftment and follow-up. **B** In vivo tumor growth. A total of 5 × 10^4^ cells were inoculated into the right thyroid lobe of the animal. Tumor growth was monitored by measuring the luminescent signal every week, and is expressed as mean (SEM) of the fold-radiance increase. Statistical analysis of the differences elicited by the treatment was estimated by two-way ANOVA (*)*p* = 0.05–0.01, (**)*p* = 0.01–0.001. **C** Violin plot showing median and quartiles of the tumor volume measured at the endpoint of the experiment. Statistical analysis of the differences between treatments was determined using a two-tailed unpaired *t* test, (*)*p* = 0.05–0.01. **D** Violin plot showing median and quartiles of the percentage of ki67-positive nuclei. Statistical analysis of the differences between treatments was determined using a two-tailed unpaired *t* test, (*)*p* = 0.05–0.01. **E** Violin plot showing median and quartiles of pERK staining intensity and percentage of positive cells (*H*-score). Statistical analysis of the differences between treatments was determined using a two-tailed unpaired *t* test, (*)*p* = 0.05–0.01

To analyze the effects of DEL-22379 on in vivo tumor progression, we used an orthotopic mouse model of ATC (Fig. 5A). A total of 5 × 10^4^ 8505c or CAL62 cells, expressing luciferase, were injected into the right lobe of the thyroid of immunocompromised nude mice, and the signal elicited by tumor cells was analyzed weekly by in vivo imaging.

We found a significant delay in primary tumor growth generated by 8505c (Fig. 5B, left panel) and CAL62 (Fig. 5B, right panel) cells in the DEL-22379-treated groups compared with animals treated with vehicle, but with greater potency in BRAF-mutant cells. In those animals inoculated with BRAF-mutant cells, the results were consistent with the reduced tumor volume, evident at the end-point of the experiment (Fig. 5C), and with the lower number of proliferating cells, which was further confirmed by Ki67 staining (Fig. 5D; tumor sections are included as Additional File 8). As a read-out of the action of DEL-22379 on ERK activation in vivo, pERK levels were measured by immunohistochemistry in tumor sections (Fig. 5E; tumor sections are included as Additional File 9), confirming the results observed in cultured cell lines.

**Fig. 6:**
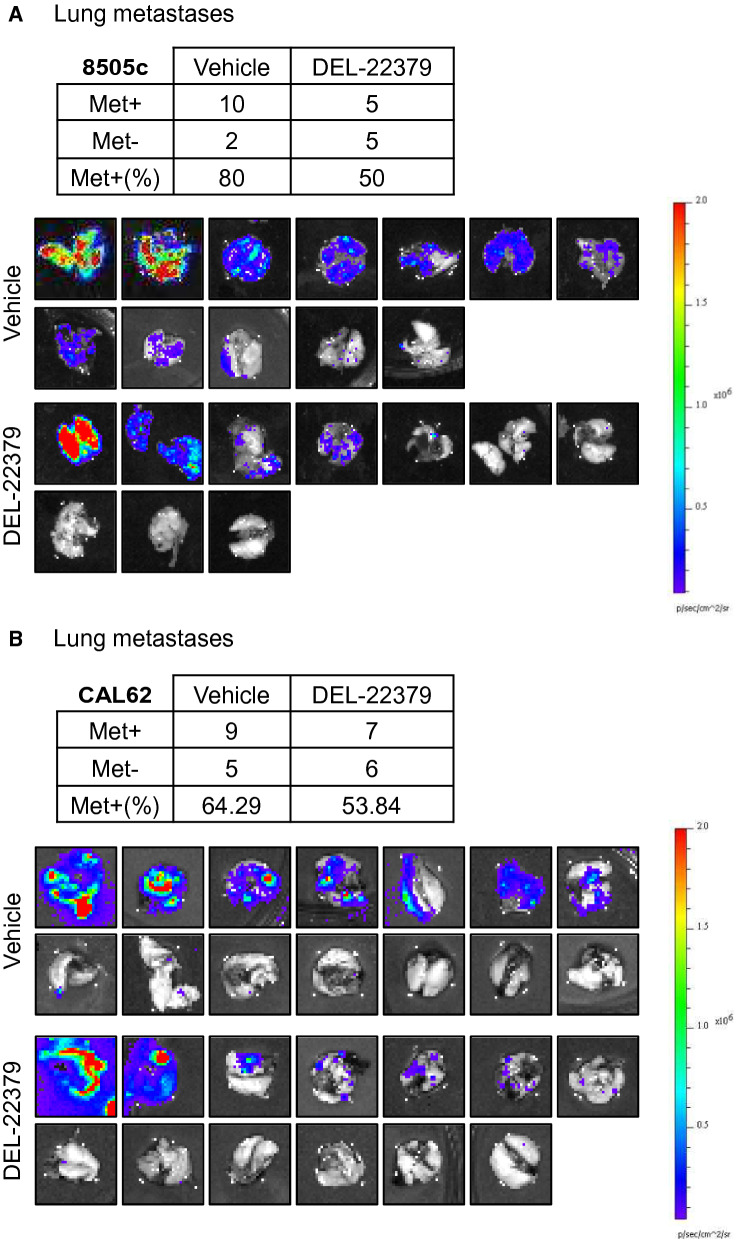
DEL-22379 effects on lung metastasis. **A** and **B** Tables represent the number of mice with (Met +) or without (Met−) lung metastasis, and the percentage of mice with positive metastasis. The complete set of lung images is provided for the interpretation of the degree of the metastatic infiltration

To assess the effect of the inhibitor on metastasis formation, we collected the lungs of the animals immediately after the end-point of the experiment and quantified the luciferase signal ex vivo. Results showed that the number of animals presenting with lung metastases was 10–30% lower in the DEL-22379-treated group than in the vehicle group (Tables in Fig. 6A and B). Furthermore, the intensity and extension of metastatic foci was strongly reduced by the drug (Fig. 6A and B).

Notably, the animals showed no signs of the toxicity commonly associated with inhibitors of the RAS-ERK pathway, such as weight loss, hand-foot skin reaction or diarrhea. A complete toxicity profile of DEL-22379 in mice has been described [18].

Overall, our results indicate that DEL-22379 effectively suppresses tumor growth of ATC-derived cell lines and impairs metastatic dissemination, especially in BRAF-mutant cells, and with no apparent signs of toxicity.

## Discussion

Thyroid cancer is typically an indolent and well-controlled disease in its differentiated form. However, dedifferentiated thyroid cancer is one of the deadliest human carcinomas [27] and no effective treatments are currently available for metastatic thyroid carcinomas. The central role of the RAS-to-ERK signaling axis for thyroid cancer onset and progression has been clearly established in the last decade [28]. Strategies designed to inhibit this pathway in advanced thyroid carcinomas, although demonstrating superiority over standard therapies, offer only modest and short-term improvements owing to, at least in part, the establishment of resistance mechanisms that allow reactivation of the pathway upon inhibition of its components [29].

An understudied feature of RAS-to-ERK signaling relates to the dimerization of the different components and its physiological implications. ERK dimerization was found to affect its subcellular localization and its ability to activate cytoplasmic but not nuclear downstream effectors [18], offering the possibility to selectively impair the cytoplasmic branch of the pathway without affecting the nuclear branch. The nuclear actions of ERK are focused more on controlling cell proliferation, whereas the cytoplasmic branch––especially through RSK––is more involved in the control of the cytoskeleton and the acquisition of metastatic properties [30]. Therefore, an appealing possibility would be to target critical drivers of the most serious manifestations of the disease without triggering resistance and with minimal secondary effects.

Our results in ATC-derived cell lines reveal that DEL-22379 displays a unique behavior not previously documented. Loss of ERK dimerization in BRAF-mutant cells elicited by the inhibitor was accompanied by a decrease in MEK and ERK phosphorylation, which would impact every process controlled by ERK. By contrast, DEL-22379 failed to impair ERK dimerization in RAS-mutant thyroid cells. This was not observed in melanoma-, CRC- or HCC-derived cell lines with RAS mutations [18]. We do not have an explanation for these discrepancies. Both BRAF and RAS mutations can promote thyroid tumorigenesis, but endow the cells with different characteristics. BRAF mutations confer stronger activation of ERK signaling, whereas RAS mutations provide oncogenic potential by constitutively activating other signaling pathways that are also related to tumor development. The gene expression and DNA methylation profiles of BRAF- and RAS-driven thyroid tumors are distinct [10], indicating that different sets of proteins are likely associated with BRAF- or RAS-driven tumors. Accordingly, if side-actions of DEL-22379 promote inactivation of one or more of the differentially-expressed proteins upstream from ERK, pathway activation could differ depending on the oncogene-associated transcriptional landscape.

In contrast to our observations in thyroid cells, no inhibition of ERK phosphorylation upon DEL-22379 pre-treatment was observed in non-thyroid cells (Additional File 3B). This could be due to the transcriptional landscape of the different cell types, although a change in the conformation and behavior of the drug associated with the metabolism of thyroid cells cannot be discarded.

Given the lack of an inhibitory effect of the drug on ERK activation in RAS-mutant ATC cells, we did not expect a therapeutic response in vitro or in vivo. However, DEL-22379 treatment of RAS-mutant cells––even at low doses––promoted changes in fibronectin expression, cell spreading and adhesion, and in in vivo tumor growth and dissemination. We believe that the most likely explanation for this is that the drug also targets the activation of other proteins in the same or different signaling pathways. Although Herrero et al. demonstrated that the specificity of the drug is high when used at a concentration of 1 µM, with only six kinases showing > 50% inhibition, the partial inhibition or activation of many other kinases was evident [18], which might explain why the drug affected different cellular processes in RAS-mutant cells in the absence of ERK inactivation. Furthermore, an effect of DEL-22379 on the tumor-associated stroma might be responsible for the delay in tumor growth and metastatic dissemination observed in mice.

While our findings have important implications for defining the mechanism of action of the molecule, it could still be of benefit as a therapy for advanced thyroid carcinomas that do not respond to conventional therapy. A completely specific inhibitor is probably unrealistic. Similar domains are shared by many proteins and kinase activity is an evolutionary success story that has been repeated in many proteins [31]. Indeed, inhibitors that were initially thought to be target-specific, such as the BRAF inhibitor sorafenib, were later found to exert their therapeutic effects through the inactivation of other proteins [32]. On the plus side, however, there was no evident toxicity in our mouse model, which is a major concern for the use of small kinase inhibitors whose administration is often discontinued in some patients or leads to treatment-associated death on some occasions. This lack of toxicity of DEL-22379 in mice might pave the way for its use with other inhibitors or compounds in combinatorial treatments. Perhaps the most promising therapy for the treatment of advanced thyroid carcinomas is based on RAS-ERK inhibition to promote cellular redifferentiation, allowing standard treatment with radioiodine [13]. Unfortunately, we did not observe a redifferentiation effect of DEL-22379 or U0126 in human tumor-derived cell lines (Additional File 10), likely precluding the use of this compound to improve radioiodine uptake.

We included samples from cells treated with the MEK inhibitor U0126 as a control for complete ERK inactivation. Although this was not the focus of the study, the use of U0126 yielded some results that are worthy of comment. Several characteristics of the ATC cells associated with an aggressive phenotype, such as expression of mesenchymal markers or cell adhesion, were increased upon MEK inhibition. The expression of important inductors of epithelial–mesenchymal transition (EMT) such as *SNAI1/2* and mesenchymal markers such as FN1 was greatly induced when ERK activation was impaired. We do not know what would be the consequences of the acquisition of a partial mesenchymal phenotype in thyroid tumor cells. According to data from The Cancer Genome Atlas (http://gepia.cancer-pku.cn/detail.php?gene=fn1), thyroid carcinomas present the highest expression of *FN1* among human carcinomas. We found that MEK inhibition further increased FN1 expression. If this benefits the tumor cell, it could represent a mechanism of resistance to the inhibition of this important signaling pathway and raises the question of whether fibronectin could represent a good therapeutic target.

These observations underscore the differential effects of the driver mutation in the different cell lines in terms of response to growth factors and subsequent signaling activation. Our findings also provide evidence of the importance of careful characterization of drugs with an intended therapeutic purpose by using cell lines that represent different types of the disease––in this case, ATC with RAS or BRAF mutations.

## Conclusions

The response of ATC-derived tumor cells to the ERK dimerization inhibitor DEL-22379 appears to depend on the driver oncogene. The mechanism of action of the drug in thyroid tumor cells differs from previous observations, impairing upstream activation of the pathway in BRAF-mutant cells, but with no effect in RAS-mutant cells. Nevertheless*,* the drug provides therapeutic benefit in delaying tumor growth and metastatic dissemination in both cell types, albeit with greater potency in BRAF-mutant cells.

## Supplementary Information

Below is the link to the electronic supplementary material.

Additional file 1. Add. Table 1.doc. List of primers. Primers used in this work, showing the target gene, sequence and orientation (PDF 34 KB)

Additional file 2. Add. Figure 1.ppt. Additional material on ERK dimerization. A. Native western blots showing an EGF dose-response assay in 8505c and CAL62 cells. ERK2 was detected to show monomer (m) and dimer (d) accumulation. B. Native western blots showing a DEL-22379 dose-response assay in 8505c and CAL62 cells. After pretreatment with DEL-22379, 10 ng/ml EGF was added and ERK2 expression was detected to show the monomeric (m) and dimeric (d) fractions (PDF 175 KB)

Additional file 3. Figure 2.ppt. DEL-22379 effects on ERK dimerization-deficient mutant. ERK dimerization in non-thyroid cell lines. A. Western blot assay showing ERK phosphorylation in 8505c cells transfected with backbone vector (pCEFL), HA-ERK2WT and HA-ERK2HL, then treated with 100 ng/ml EGF after pre-treatment with vehicle (DMSO), 10 µM DEL-22379 or 10 µM U0126 B. Native- (upper panel) and SDS- (lower panels) PAGE western blots showing the effects of DEL-22379 and U0126 on ERK dimerization and phosphorylation in HEK293T and A375P cell lines. For ERK2 protein analysis, the upper band in the gels corresponds to the monomeric (m) form and the lower band to the dimeric (d) form (PDF 127 KB)

Additional file 5. Add. Figure 3.ppt. pERK regulation by DEL22379 and U0126. Western blot of 8505c and OCUT2 cells showing ERK phosphorylation state upon EGF (100 ng/ml) or 10% FBS treatment for 5 min in the presence of 10 µM DEL-22379 (D) or U0126 (U0) (PDF 110 KB)

Additional file 6. Add. Figure 4.ppt. Analysis of RNA sequencing. ALL PANELS. DEL-22379 1 µM (D1) and 10 µM (D10) A. Principal component analysis of individual samples. B. Sample-to-sample heatmap. C. Table showing the number of under/overexpressed genes with p adjusted value <0.01 and shrunken log Fold Change >1.5 or <-1.5. D and E. Venn diagrams showing the number of overlapping under (D) and overexpressed (E) genes among treatments and cell lines. F and G. Gene enrichment analysis of differentially expressed genes in 8505c (F) and CAL62 (G) cells (PDF 743 KB)

Additional file 7. Table 2.xlsx. List of differentially expressed genes. Complete list of differentially-expressed genes used in Additional file 7 D–G. Genes were filtered by p adjusted value <0.01 and shrunken log Fold Change >1.5 or <-1.5, except for genes regulated by DEL-22379 at 1 µM, where a less restrictive criterion was used. Names of genes in blue and red were, respectively, found under and overexpressed (XLSX 534 KB)

Additional file 8. Add. Figure 5.ppt. Ki67 IHCs of tumor mice. Representative immunohistochemistry image of primary tumors stained for Ki67 from each mouse (PDF 382 KB)

Additional file 9. Add. Figure 6.ppt. pERK IHCs of tumor mice. Representative immunohistochemistry image of primary tumors stained for pERK from each mouse (PDF 587 KB)

Additional file 10. Add. Figure 7. ppt. DEL-22379 effects on cellular differentiation. mRNA levels of SLC5A5 after 48 h in the presence of 10% FBS plus 1 µM (D1), 5 µM (D5) or 10 µM (D10) DEL-22379, 10 µM U0126 or vehicle (DMSO), estimated by RT-qPCR. Results are expressed as mean (SD) of 1 experiment performed by triplicate. A. BRAF-mutant cells. B. RAS-mutant cells. C. BRAF-mutant cells including MDCK-hNIS as a positive control for SLC5A5 expression (PDF 42 KB)

Additional file 4. Migration.avi. Time-lapse wound healing assay. A representative video from each condition in Fig.3D is shown. (AVI 7020 KB)

Supplementary file11 (AVI 6766 KB)

Supplementary file12 (AVI 8712 KB)

Supplementary file13 (AVI 7132 KB)

Supplementary file14 (AVI 11810 KB)

Supplementary file15 (AVI 13314 KB)

Supplementary file16 (AVI 11966 KB)

Supplementary file17 (AVI 11773 KB)

Supplementary file18 (AVI 5061 KB)

Supplementary file19 (AVI 5041 KB)

Supplementary file20 (AVI 5705 KB)

Supplementary file21 (AVI 4381 KB)

Supplementary file22 (AVI 3942 KB)

Supplementary file23 (AVI 3913 KB)

Supplementary file24 (AVI 4584 KB)

Supplementary file25 (AVI 4095 KB)

## Data Availability

RNA-seq data are available in GSE190711. The datasets supporting the conclusions of this article are included within the article and its additional files and are available from the corresponding author on reasonable request.

## Abbreviations

PTC: Papillary thyroid carcinoma
FTC: Follicular thyroid carcinoma
WDTC: Well-differentiated thyroid carcinomas
ATC: Anaplastic thyroid carcinoma
PDTC: Poorly differentiated thyroid carcinoma
DMEM: Dulbecco’s Modified Eagle’s Medium
FBS: Fetal bovine serum
EGF: Epidermal growth factor
BSA: Bovine serum albumin
PFA: Paraformaldehyde
DAPI: 4′,6-Diamidino-2-phenylindole
HCC: Hepatocellular carcinoma
CRC: Colorectal carcinoma
FAs: Focal adhesions
EMT: Epithelial-mesenchymal transition
