# Supplementary material for: Inhibiting ERK dimerization ameliorates BRAF-driven anaplastic thyroid cancer
Source: Cell Mol Life Sci. 2022 Sep 3;79(9):504. doi: 10.1007/s00018-022-04530-9 (PMC9440884; doi:10.1007/s00018-022-04530-9)
Supplement: Supplementary file 1 — Additional file 1. Add. Table 1.doc. List of primers. Primers used in this work, showing the target gene, sequence and orientation (PDF 34 KB) [file 18_2022_4530_MOESM1_ESM.pdf]

Additional file 1. Table S1

GENE_ORIENTATION	SEQUENCE
FOS_Fwd	5'-TGTGAAGACCATGACAGGAGG-3'
FOS_Rv	5'-TTGGTCTGTCTCCGCTTGG-3'
JUN_Fwd	5'-GCGGACCTTATGGCTACAGT-3'
JUN_Rv	5'- CCCGTTGCTGGACTGGATTA-3'
EGR1_Fwd	5'-TGACCGCAGAGTCTTTTCCTG-3'
EGR1_Rv	5'-GGTCATGCTCACTAGGCCAC-3'
SNAI1_Fwd	5'- GCCTAGCGAGTGGTTCTTCT-3'
SNAI1_Rv	5'-GCCAGGACAGAGTCCCAGAT-3'
SNAI2_Fwd	5'- TTCCAGACCCTGGTTGCTTC-3'
SNAI2_Rv	5'-ATTGCGTCACTCAGTGTGCT-3'
ZEB1_Fwd	5'-CAGAGGATGACCTGCCAACA-3'
ZEB1_Rv	5'-CTCGCATTCATCATCTTTTACTGT-3'
FN1_Fwd	5'-TGCAAGCCCATAGCTGAGAA-3'
FN1_Rv	5'-AGATGCACTGGAGCAGGTTT-3'
VIM_Fwd	5'-AAGGCGAGGAGAGCAGGATTT-3'
VIM_Rv	5'-CGTGATGCTGAGAAGTTTCGT -3'
CDH2_Fwd	5'-TGTGGACAGGATTGTGGGTG-3'
CDH2_Rv	5'-GGCTCAAGTCATAGTCCTGGT-3'
CTNNB1_Fwd	5'-GCCATTTTAAGCCTCTCGGTC-3'
CTNNB1_Rv	5'-CATCAAATCAGCTTGAGTAGCC-3'
TBP_Fwd	5'-GCAAGGGTTTCTGGTTTGCC-3'
TBP_Rv	5'-GGGTCAGTCCAGTGCCATAA-3'
SLC5A5_Fwd	5'-CTCATCCTGAACCAAGTGAC-3'
SLC5A5_Rv	5'-GAACACATCAGTCCAGACCA-3'
