# Supplementary figures and images for: Inhibiting ERK dimerization ameliorates BRAF-driven anaplastic thyroid cancer
Source: Cell Mol Life Sci. 2022 Sep 3;79(9):504. doi: 10.1007/s00018-022-04530-9 (PMC9440884; doi:10.1007/s00018-022-04530-9)

**A**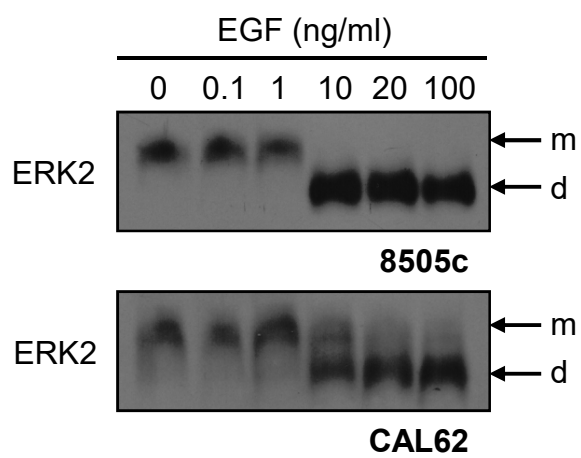**B**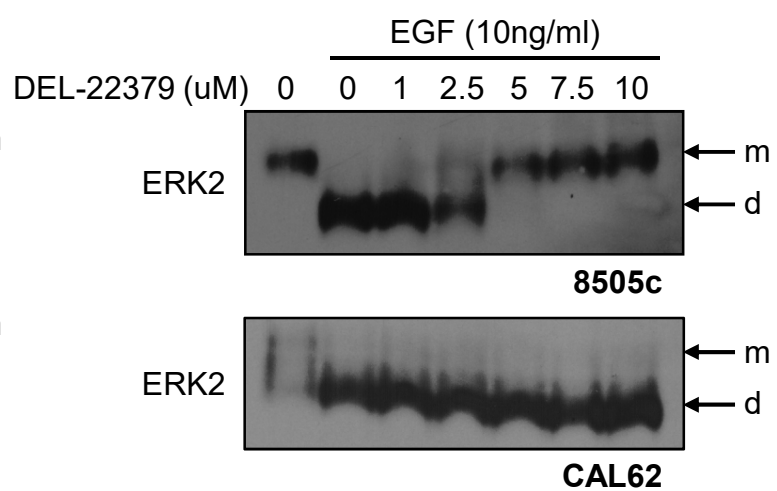

Supplement: Supplementary file 2 — Additional file 2. Add. Figure 1.ppt. Additional material on ERK dimerization. A. Native western blots showing an EGF dose-response assay in 8505c and CAL62 cells. ERK2 was detected to show monomer (m) and dimer (d) accumulation. B. Native western blots showing a DEL-22379 dose-response assay in 8505c and CAL62 cells. After pretreatment with DEL-22379, 10 ng/ml EGF was added and ERK2 expression was detected to show the monomeric (m) and dimeric (d) fractions (PDF 175 KB) [file 18_2022_4530_MOESM2_ESM.pdf]

**A**

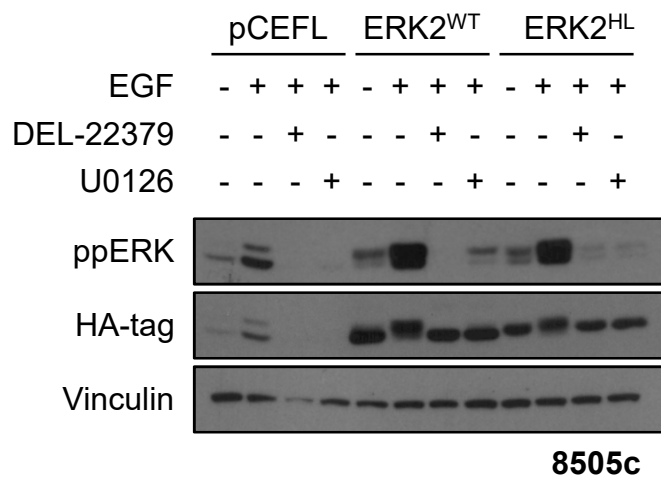

**B**

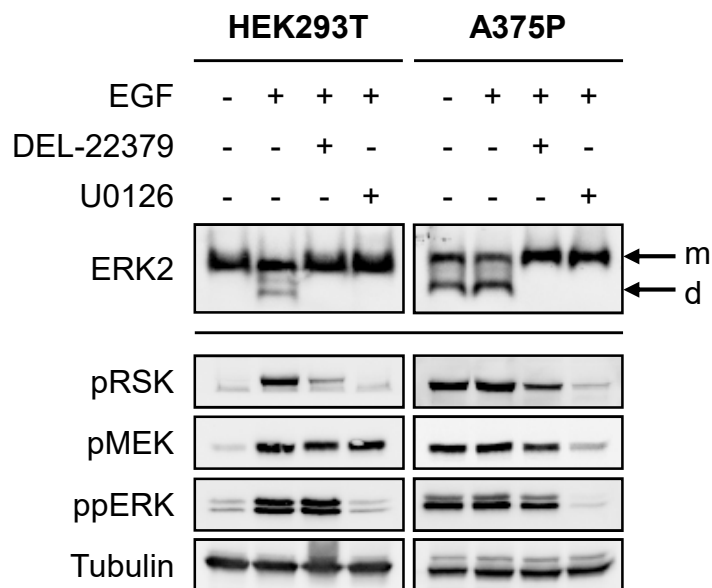

Supplement: Supplementary file 3 — Additional file 3. Figure 2.ppt. DEL-22379 effects on ERK dimerization-deficient mutant. ERK dimerization in non-thyroid cell lines. A. Western blot assay showing ERK phosphorylation in 8505c cells transfected with backbone vector (pCEFL), HA-ERK2WT and HA-ERK2HL, then treated with 100 ng/ml EGF after pre-treatment with vehicle (DMSO), 10 µM DEL-22379 or 10 µM U0126 B. Native- (upper panel) and SDS- (lower panels) PAGE western blots showing the effects of DEL-22379 and U0126 on ERK dimerization and phosphorylation in HEK293T and A375P cell lines. For ERK2 protein analysis, the upper band in the gels corresponds to the monomeric (m) form and the lower band to the dimeric (d) form (PDF 127 KB) [file 18_2022_4530_MOESM3_ESM.pdf]

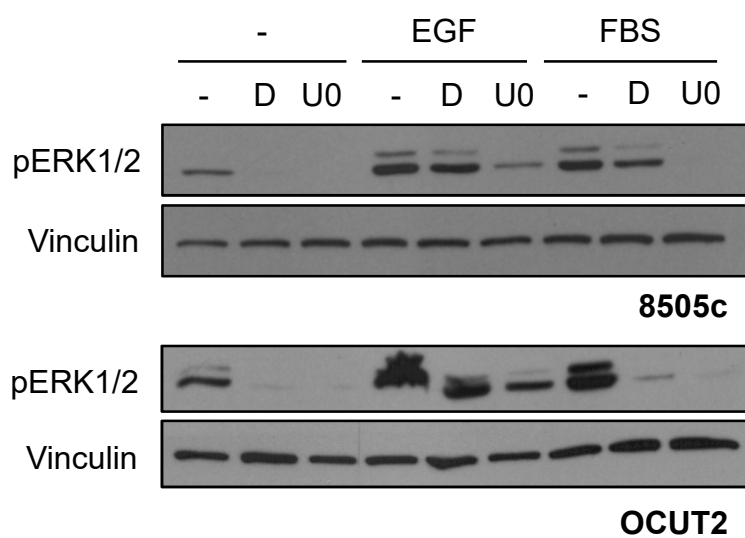

Supplement: Supplementary file 4 — Additional file 5. Add. Figure 3.ppt. pERK regulation by DEL22379 and U0126. Western blot of 8505c and OCUT2 cells showing ERK phosphorylation state upon EGF (100 ng/ml) or 10% FBS treatment for 5 min in the presence of 10 µM DEL-22379 (D) or U0126 (U0) (PDF 110 KB) [file 18_2022_4530_MOESM4_ESM.pdf]

---

**8505c**

---

Vehicle

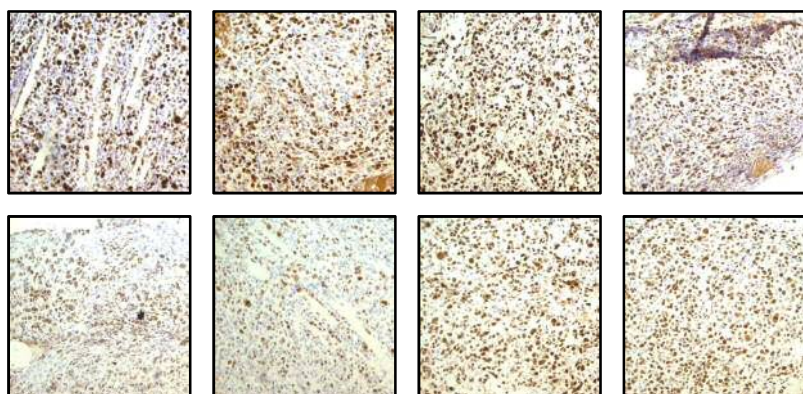

DEL-22379

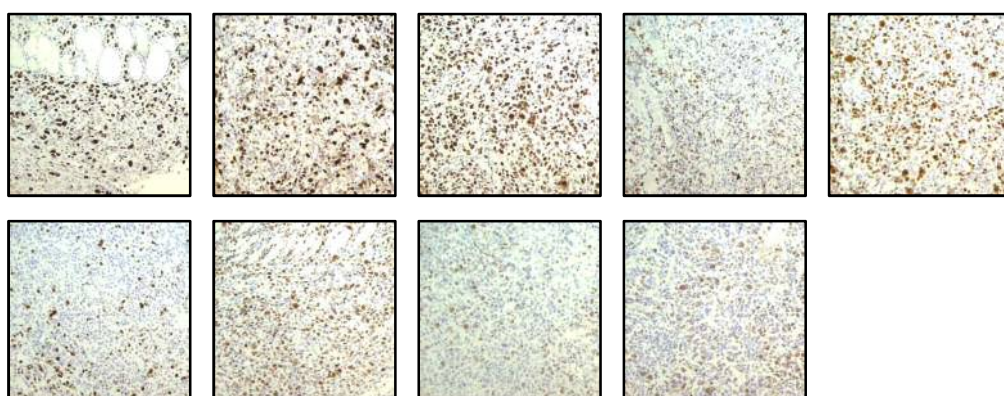

---

**CAL62**

---

Vehicle

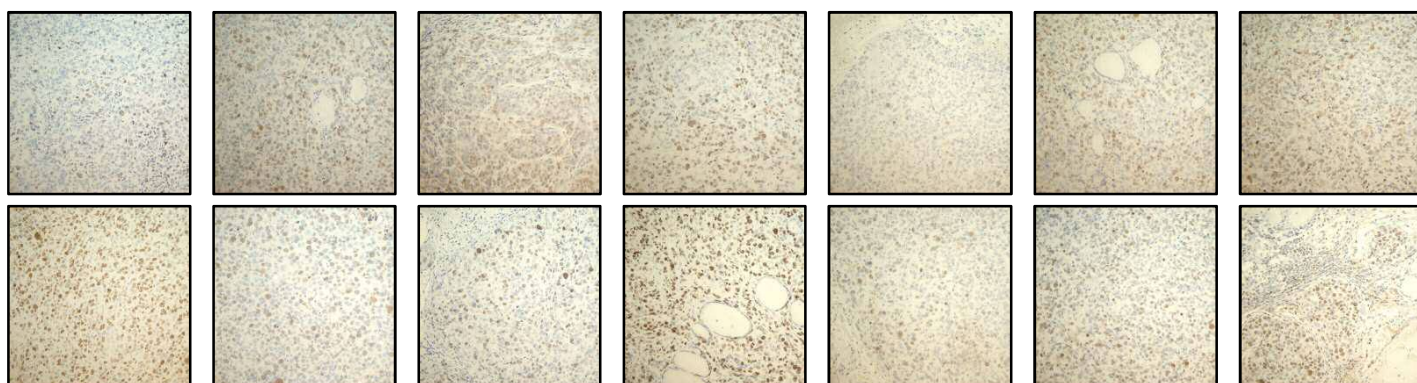

DEL-22379

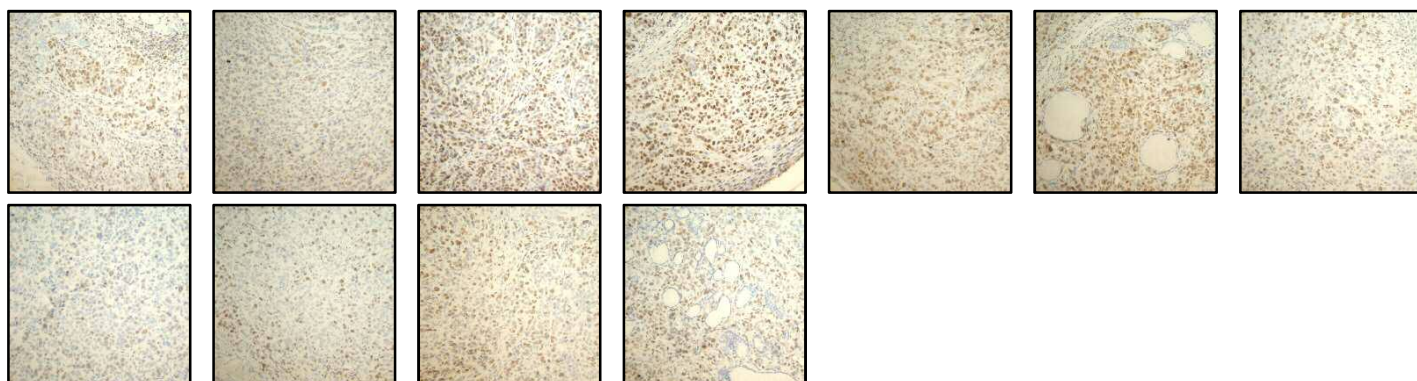

Supplement: Supplementary file 7 — Additional file 8. Add. Figure 5.ppt. Ki67 IHCs of tumor mice. Representative immunohistochemistry image of primary tumors stained for Ki67 from each mouse (PDF 382 KB) [file 18_2022_4530_MOESM7_ESM.pdf]

## Vehicle

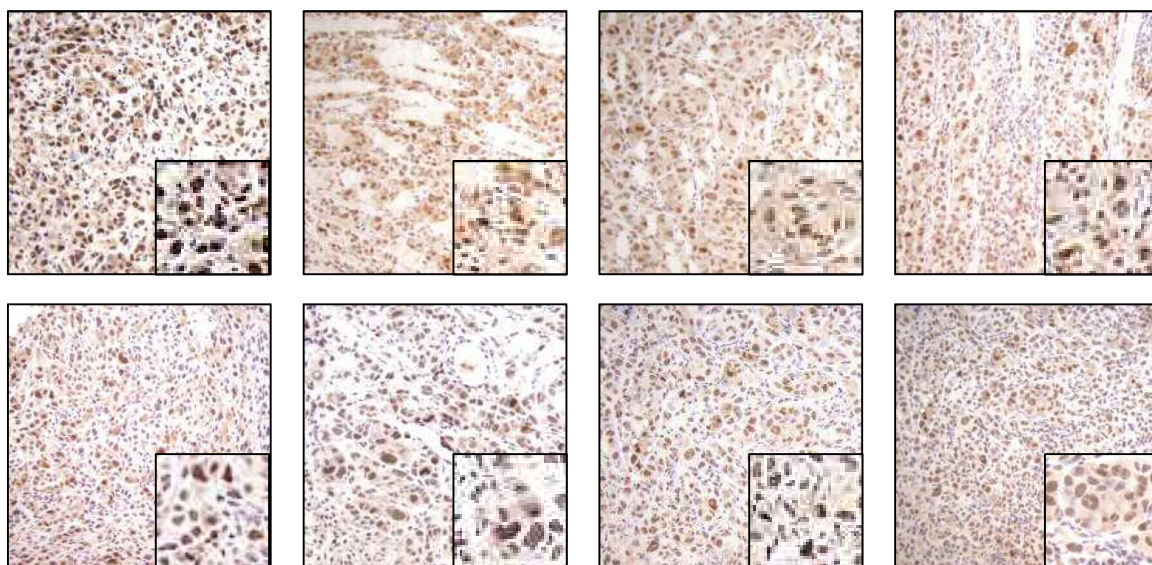

## DEL-22379

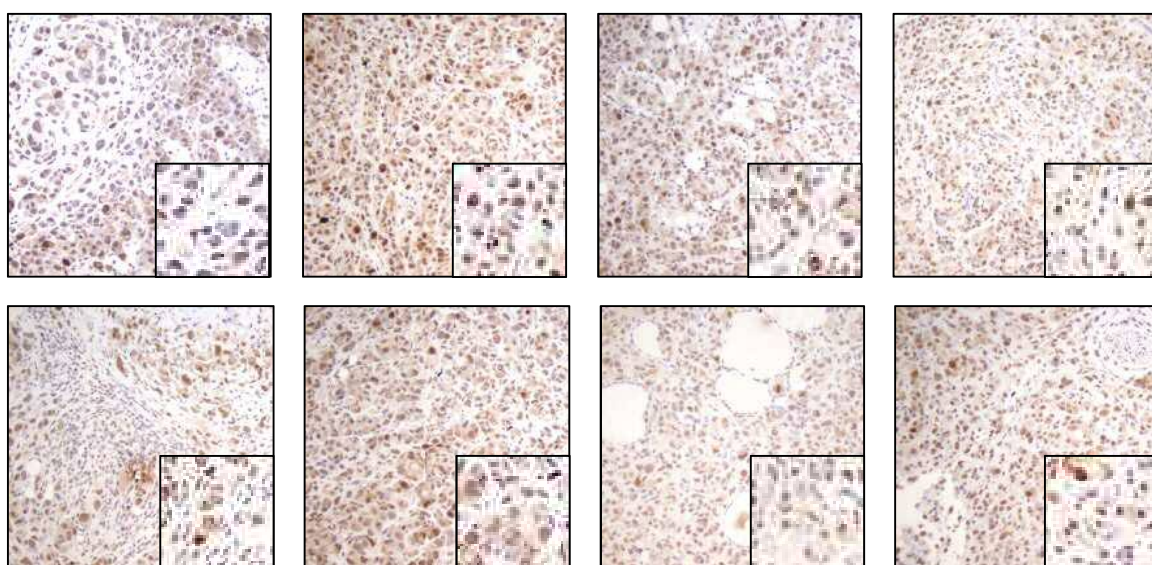

Vehicle

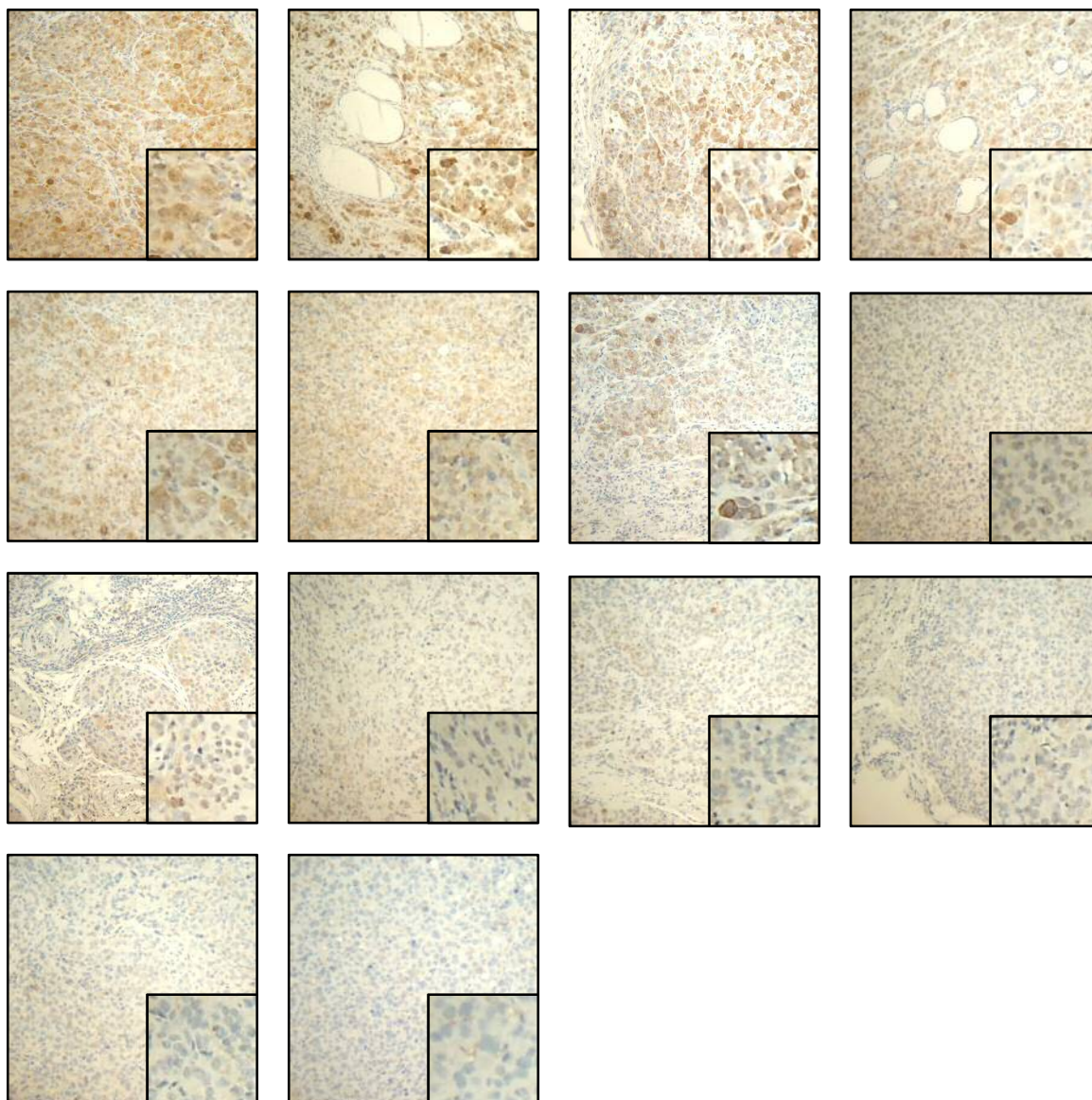

DEL-22379

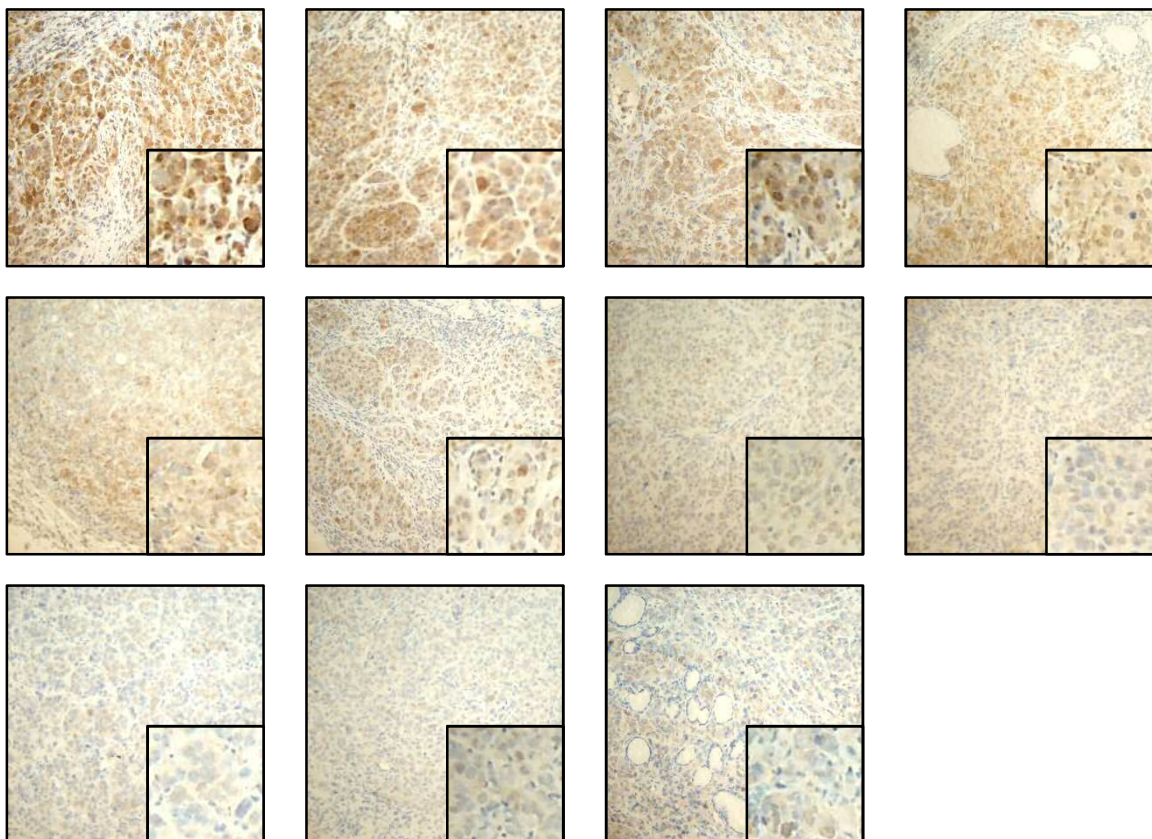

Supplement: Supplementary file 8 — Additional file 9. Add. Figure 6.ppt. pERK IHCs of tumor mice. Representative immunohistochemistry image of primary tumors stained for pERK from each mouse (PDF 587 KB) [file 18_2022_4530_MOESM8_ESM.pdf]

**A**

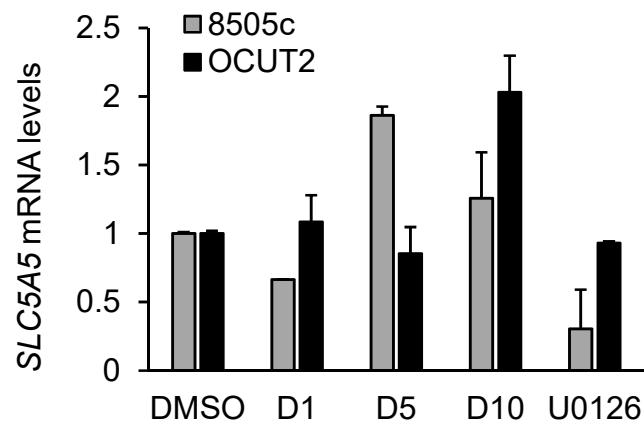

**B**

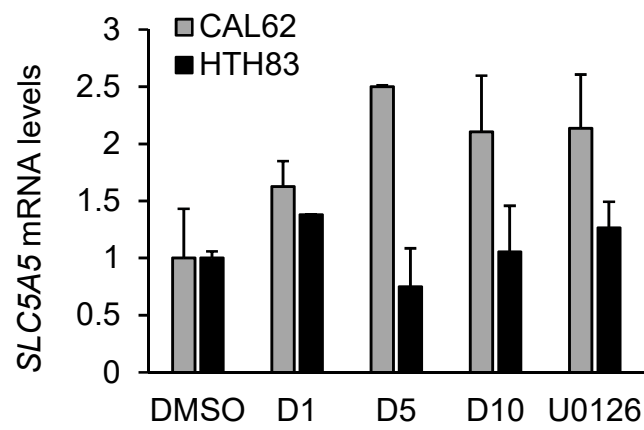

**C**

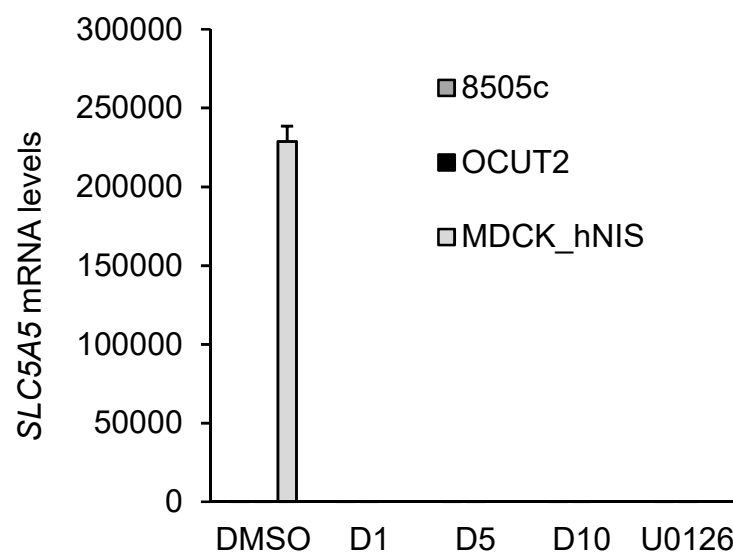

Supplement: Supplementary file 9 — Additional file 10. Add. Figure 7. ppt. DEL-22379 effects on cellular differentiation. mRNA levels of SLC5A5 after 48 h in the presence of 10% FBS plus 1 µM (D1), 5 µM (D5) or 10 µM (D10) DEL-22379, 10 µM U0126 or vehicle (DMSO), estimated by RT-qPCR. Results are expressed as mean (SD) of 1 experiment performed by triplicate. A. BRAF-mutant cells. B. RAS-mutant cells. C. BRAF-mutant cells including MDCK-hNIS as a positive control for SLC5A5 expression (PDF 42 KB) [file 18_2022_4530_MOESM9_ESM.pdf]
