# Supplementary material for: Inhibiting ERK dimerization ameliorates BRAF-driven anaplastic thyroid cancer
Source: Cell Mol Life Sci. 2022 Sep 3;79(9):504. doi: 10.1007/s00018-022-04530-9 (PMC9440884; doi:10.1007/s00018-022-04530-9)
Supplement: Supplementary file 5 — Additional file 6. Add. Figure 4.ppt. Analysis of RNA sequencing. ALL PANELS. DEL-22379 1 µM (D1) and 10 µM (D10) A. Principal component analysis of individual samples. B. Sample-to-sample heatmap. C. Table showing the number of under/overexpressed genes with p adjusted value <0.01 and shrunken log Fold Change >1.5 or <-1.5. D and E. Venn diagrams showing the number of overlapping under (D) and overexpressed (E) genes among treatments and cell lines. F and G. Gene enrichment analysis of differentially expressed genes in 8505c (F) and CAL62 (G) cells (PDF 743 KB) [file 18_2022_4530_MOESM5_ESM.pdf]

A

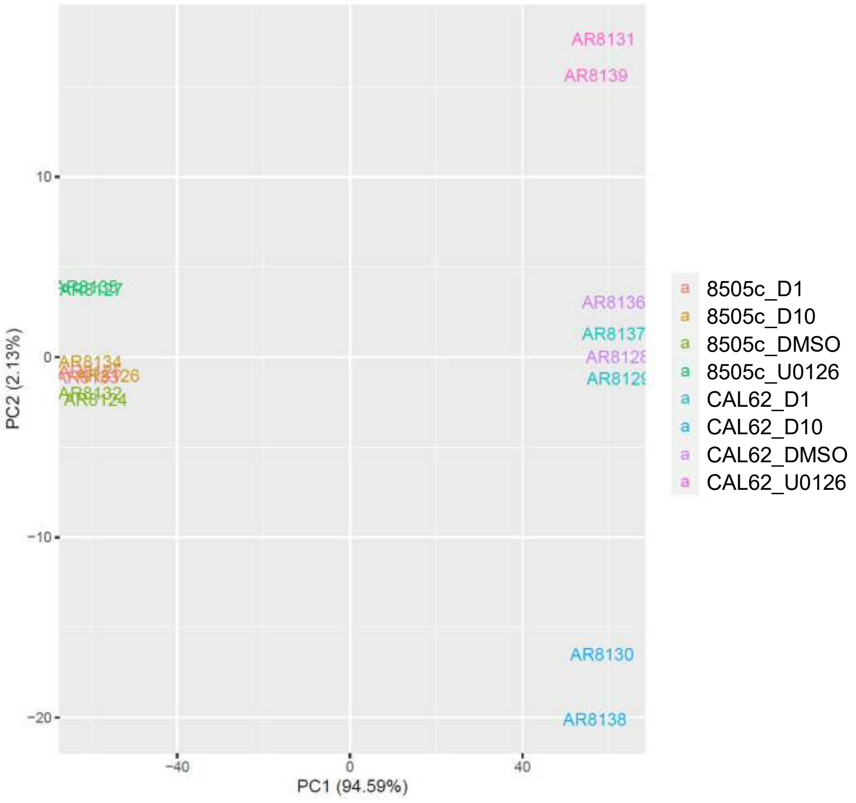

B

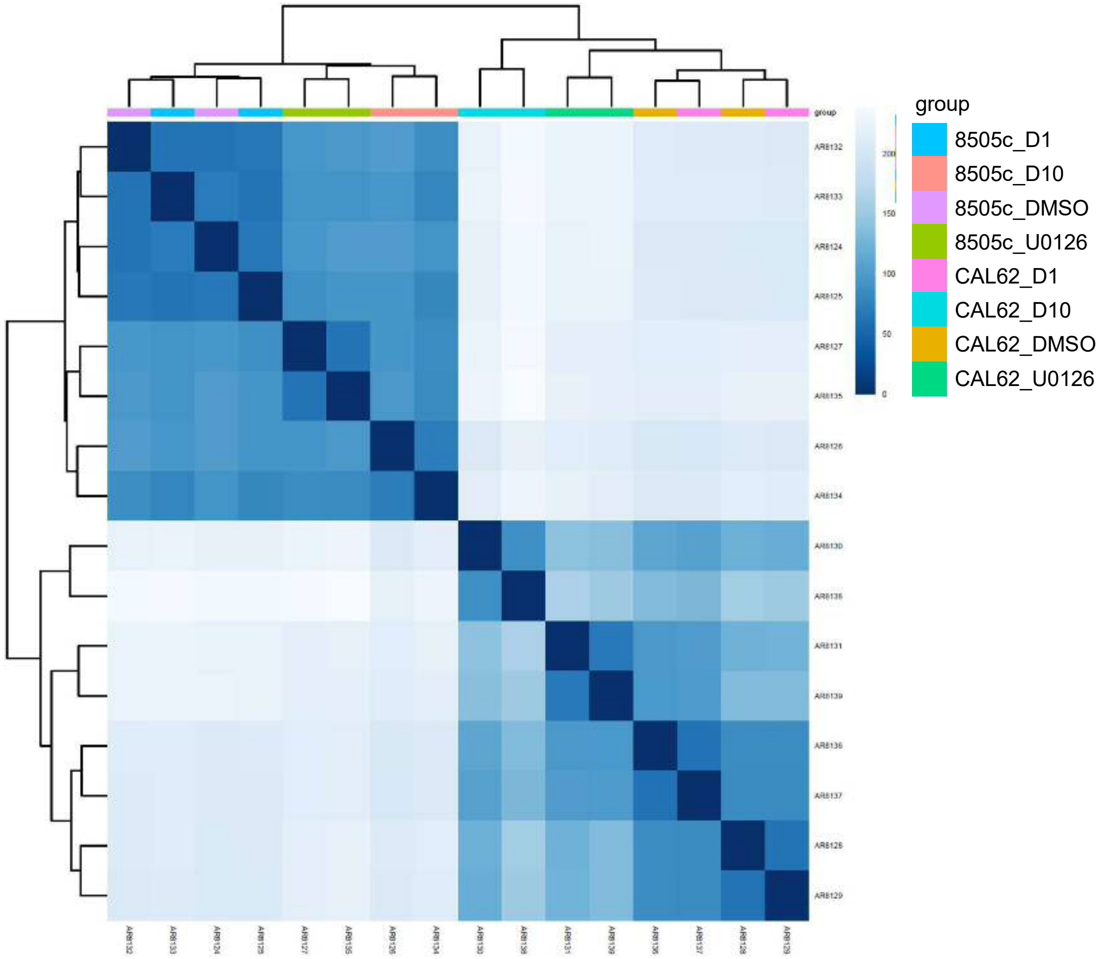

Additional file 6, Zaballos et al.

C

	8505c	CAL62
D1	(5/3)	(0/1)
D10	(152/357)	(604/886)
U0126	(243/421)	(636/581)

Under/Overexpressed genes

D

Underexpressed genes

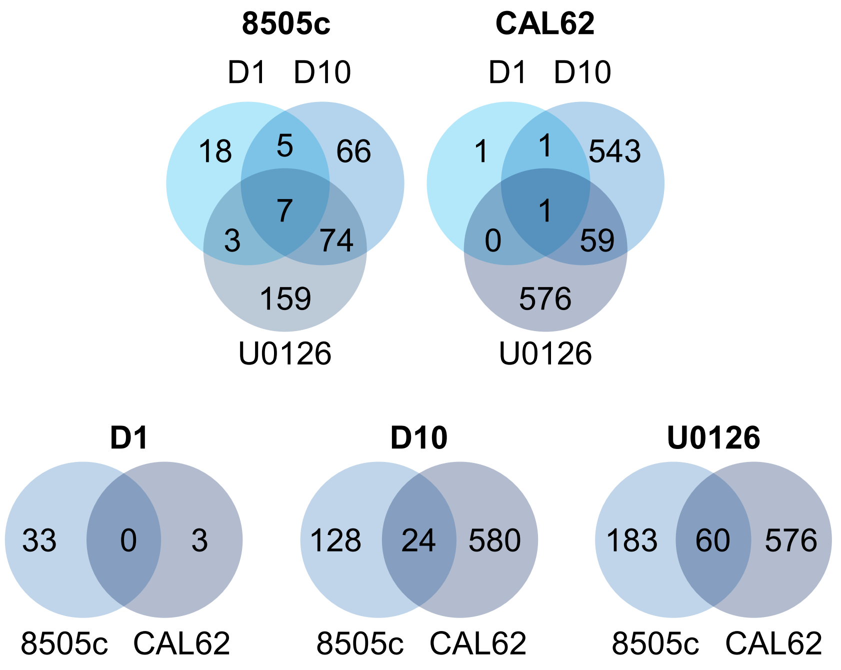

E

Overexpressed genes

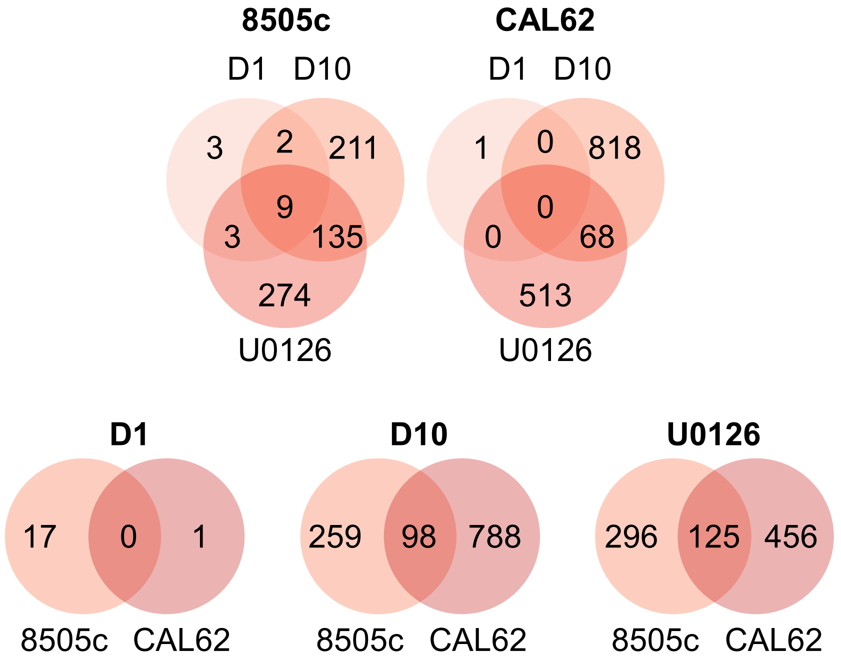

Additional file 6, Zaballos et al.

F

8505c_DEL-22379 1μM. Gene enrichment analysis. Underexpressed genes

GO:BP		stats				
Term name	Term ID	P_{adj} ↑	$-\log_{10}(P_{adj})$	T	Q	T∩Q
response to cytokine	GO:0034097	3.082×10^{-4}		1226	29	12
cellular response to chemical stimulus	GO:0070887	4.252×10^{-3}		3393	29	17
extracellular matrix organization	GO:0030198	5.062×10^{-3}		401	29	7
regulation of epithelial cell differentiation	GO:0030856	1.139×10^{-2}		164	29	5
regulation of cell death	GO:0010941	1.150×10^{-2}		1720	29	12

1 to 5 of 5 Page 1 of 1

8505c_DEL-22379 1μM. Gene enrichment analysis. Overexpressed genes

GO:BP		stats				
Term name	Term ID	P_{adj}	$-\log_{10}(P_{adj})$	T	Q	T∩Q
cholesterol biosynthetic process	GO:0006695	6.813×10^{-4}		74	16	4
steroid biosynthetic process	GO:0006694	8.460×10^{-4}		196	16	5

1 to 2 of 2 Page 1 of 1

8505c_DEL-22379 10μM. Gene enrichment analysis. Underexpressed genes

GO:MF		stats				
Term name	Term ID	P_{adj}	$-\log_{10}(P_{adj})$	T	Q	T∩Q
cytokine binding	GO:0019955	9.532×10^{-3}		138	113	7
growth factor receptor binding	GO:0070851	1.199×10^{-2}		143	113	7
small GTPase binding	GO:0031267	3.815×10^{-2}		298	113	9

1 to 3 of 3 Page 1 of 1

GO:BP		stats				
Term name	Term ID	P_{adj}	$-\log_{10}(P_{adj})$	T	Q	T∩Q
angiogenesis	GO:0001525	1.097×10^{-5}		617	111	19
cellular response to chemical stimulus	GO:0070887	1.342×10^{-4}		3393	111	45
cell communication	GO:0007154	6.898×10^{-4}		6829	111	68
chemotaxis	GO:0006935	8.228×10^{-4}		653	111	17
transmembrane receptor protein tyrosine kinase signaling pathway	GO:0007169	1.441×10^{-3}		760	111	18
signaling	GO:0023052	1.614×10^{-3}		6810	111	67
cell differentiation	GO:0030154	4.364×10^{-3}		4350	111	49
cell adhesion	GO:0007155	1.718×10^{-2}		1492	111	24
MAPK cascade	GO:0000165	2.590×10^{-2}		935	111	18
positive regulation of inflammatory response	GO:0050729	4.818×10^{-2}		142	111	7

1 to 10 of 10 Page 1 of 1

GO:CC		stats					
Term name	Term ID	P_{adj}	$-\log_{10}(P_{adj})$	T	Q	T∩Q	U
plasma membrane	GO:0005886	9.283×10^{-3}		5681	119	57	18960

1 to 1 of 1 Page 1 of 1

8505c_DEL-22379 10μM. Gene enrichment analysis. Overexpressed genes

GO:BP		stats				
Term name	Term ID	P_{adj}	$-\log_{10}(P_{adj})$	T	Q	T∩Q
cellular response to extracellular stimulus	GO:0031668	2.063×10^{-2}		248	251	14
lipid metabolic process	GO:0006629	4.873×10^{-2}		1457	251	40

1 to 2 of 2 Page 1 of 1

GO:CC		stats				
Term name	Term ID	P_{adj}	$-\log_{10}(P_{adj})$	T	Q	T∩Q
plasma membrane	GO:0005886	1.504×10^{-2}		5681	272	112

1 to 1 of 1 Page 1 of 1

F

8505c_U0126. Gene enrichment analysis. Underexpressed genes

GO:MF		stats				
Term name	Term ID	p_{adj}	$-\log_{10}(p_{adj})$ 0 … ≤16	T	Q	T∩Q
signaling receptor binding	GO:0005102	4.076×10^{-4}		1672	176	37
cytokine binding	GO:0019955	2.793×10^{-2}		138	176	8

1 to 2 of 2 Page 1 of 1

GO:BP		stats				
Term name	Term ID	p_{adj}	$-\log_{10}(p_{adj})$ 0 … ≤16	T	Q	T∩Q
response to growth factor	GO:0070848	3.693×10^{-7}		759	174	29
angiogenesis	GO:0001525	4.506×10^{-7}		617	174	26
cell communication	GO:0007154	5.138×10^{-6}		6829	174	104
cell population proliferation	GO:0008283	5.586×10^{-6}		2054	174	48
cellular response to chemical stimulus	GO:0070887	1.122×10^{-5}		3393	174	65
cell migration	GO:0016477	1.977×10^{-5}		1649	174	41
signaling	GO:0023052	2.613×10^{-5}		6810	174	102
epithelial cell differentiation	GO:0030855	9.391×10^{-5}		798	174	26
regulation of kinase activity	GO:0043549	1.159×10^{-4}		921	174	28
response to fibroblast growth factor	GO:0071774	5.362×10^{-4}		153	174	11
chemotaxis	GO:0006935	2.558×10^{-3}		653	174	21
regulation of MAP kinase activity	GO:0043405	4.620×10^{-3}		316	174	14
transmembrane receptor protein tyrosine kinase signaling pathway	GO:0007169	7.832×10^{-3}		760	174	22
mesenchyme development	GO:0060485	9.816×10^{-3}		291	174	13

1 to 14 of 14 Page 1 of 1

GO:CC		stats				
Term name	Term ID	p_{adj}	$-\log_{10}(p_{adj})$ 0 … ≤16	T	Q	T∩Q
plasma membrane	GO:0005886	5.212×10^{-4}		5681	180	84

1 to 1 of 1 Page 1 of 1

8505c_U0126. Gene enrichment analysis. Overexpressed genes

GO:MF		stats				
Term name	Term ID	p_{adj}	$-\log_{10}(p_{adj})$ 0 … ≤16	T	Q	T∩Q
extracellular matrix structural constituent	GO:0005201	9.692×10^{-3}		171	349	13

1 to 1 of 1 Page 1 of 1

GO:BP		stats				
Term name	Term ID	p_{adj}	$-\log_{10}(p_{adj})$ 0 … ≤16	T	Q	T∩Q
lipid metabolic process	GO:0006629	6.481×10^{-4}		1457	327	54
extracellular matrix organization	GO:0030198	8.947×10^{-3}		401	327	22
regulation of intracellular signal transduction	GO:1902531	9.437×10^{-3}		1842	327	60

1 to 3 of 3 Page 1 of 1

GO:CC		stats				
Term name	Term ID	p_{adj}	$-\log_{10}(p_{adj})$ 0 … ≤16	T	Q	T∩Q
extracellular matrix	GO:0031012	1.612×10^{-4}		562	346	29
cytoplasm	GO:0005737	3.524×10^{-2}		11911	346	250

1 to 2 of 2 Page 1 of 1

G

CAL62_DEL-22379 10μM. Gene enrichment analysis. Underexpressed genes

GO:MF		stats				
Term name	Term ID	p_{adj}	$-\log_{10}(p_{adj})$	T	Q	T∩Q
calcium ion binding	GO:0005509	1.667×10^{-5}		721	458	46
extracellular matrix structural constituent	GO:0005201	4.345×10^{-2}		171	458	14

1 to 2 of 2 Page 1 of 1

GO:BP		stats				
Term name	Term ID	p_{adj}	$-\log_{10}(p_{adj})$	T	Q	T∩Q
cell adhesion	GO:0007155	2.582×10^{-9}		1492	437	82
cell junction organization	GO:0034330	2.333×10^{-3}		722	437	40
stem cell development	GO:0048864	1.352×10^{-2}		84	437	11
mesenchymal cell development	GO:0014031	1.352×10^{-2}		84	437	11
response to transforming growth factor beta	GO:0071559	1.534×10^{-2}		262	437	20
extracellular matrix organization	GO:0030198	3.931×10^{-2}		401	437	25

1 to 6 of 6 Page 1 of 1

GO:CC		stats				
Term name	Term ID	p_{adj}	$-\log_{10}(p_{adj})$	T	Q	T∩Q
extracellular matrix	GO:0031012	3.657×10^{-4}		562	459	34
endoplasmic reticulum lumen	GO:0005788	6.627×10^{-4}		307	459	23
plasma membrane	GO:0005886	2.673×10^{-3}		5681	459	181

1 to 3 of 3 Page 1 of 1

CAL62_DEL-22379 10μM. Gene enrichment analysis. Overexpressed genes

GO:MF		stats				
Term name	Term ID	p_{adj}	$-\log_{10}(p_{adj})$	T	Q	T∩Q
DNA-binding transcription activator activity	GO:0001216	3.760×10^{-4}		455	627	37
signaling receptor binding	GO:0005102	4.094×10^{-4}		1672	627	93
protein kinase binding	GO:0019901	2.303×10^{-2}		673	627	43
mitogen-activated protein kinase binding	GO:0051019	4.002×10^{-2}		32	627	7

1 to 4 of 4 Page 1 of 1

GO:BP		stats				
Term name	Term ID	p_{adj}	$-\log_{10}(p_{adj})$	T	Q	T∩Q
cell differentiation	GO:0030154	5.087×10^{-16}		4350	604	243
cellular response to chemical stimulus	GO:0070887	1.630×10^{-10}		3393	604	188
cell population proliferation	GO:0008283	1.025×10^{-6}		2054	604	120
cell communication	GO:0007154	5.139×10^{-6}		6829	604	298
signaling	GO:0023052	1.639×10^{-5}		6810	604	295
response to cytokine	GO:0034097	2.782×10^{-5}		1226	604	79
positive regulation of transcription by RNA polymerase II	GO:0045944	1.016×10^{-4}		1197	604	76
cell adhesion	GO:0007155	4.490×10^{-4}		1492	604	87
regulation of transport	GO:0051049	1.149×10^{-3}		1809	604	99
protein kinase B signaling	GO:0043491	3.084×10^{-3}		286	604	27

1 to 10 of 10 Page 1 of 1

GO:CC		stats				
Term name	Term ID	p_{adj}	$-\log_{10}(p_{adj})$	T	Q	T∩Q
extracellular region	GO:0005576	2.091×10^{-3}		4567	638	202
cell periphery	GO:0071944	2.480×10^{-2}		6169	638	253

1 to 2 of 2 Page 1 of 1

G

CAL62_U0126. Gene enrichment analysis. Underexpressed genes

GO:MF		stats				
Term name	Term ID	P_{adj}	$-\log_{10}(P_{adj})$	T	Q	TnQ
DNA helicase activity	GO:0003678	2.120×10^{-16}		76	527	24
ATPase activity	GO:0016887	1.767×10^{-14}		488	527	53
DNA replication origin binding	GO:0003688	1.304×10^{-10}		23	527	12
microtubule binding	GO:0008017	4.548×10^{-9}		271	527	32
DNA binding	GO:0003677	1.460×10^{-6}		2486	527	119
damaged DNA binding	GO:0003684	1.220×10^{-3}		66	527	11
cytoskeletal protein binding	GO:0008092	1.851×10^{-3}		996	527	54
kinase binding	GO:0019900	1.206×10^{-2}		752	527	42

1 to 8 of 8 Page 1 of 1

GO:BP		stats				
Term name	Term ID	P_{adj}	$-\log_{10}(P_{adj})$	T	Q	TnQ
cell cycle	GO:0007049	8.800×10^{-71}		1902	521	211
chromosome segregation	GO:0007059	7.300×10^{-51}		334	521	83
organelle fission	GO:0048285	7.402×10^{-51}		497	521	98
DNA replication	GO:0006260	1.992×10^{-47}		286	521	75
cellular response to DNA damage stimulus	GO:0006974	4.570×10^{-31}		888	521	102
DNA repair	GO:0006281	4.746×10^{-30}		576	521	81
microtubule cytoskeleton organization	GO:0000226	5.101×10^{-16}		613	521	64

1 to 7 of 7 Page 1 of 1

GO:CC		stats				
Term name	Term ID	P_{adj}	$-\log_{10}(P_{adj})$	T	Q	TnQ
chromosome	GO:0005694	4.149×10^{-37}		2071	539	170
nucleus	GO:0005634	6.288×10^{-16}		7592	539	315
cytoskeleton	GO:0005856	2.040×10^{-6}		2332	539	113
organelle	GO:0043226	4.898×10^{-4}		14008	539	444

1 to 4 of 4 Page 1 of 1

CAL62_U0126. Gene enrichment analysis. Overexpressed genes

GO:MF		stats				
Term name	Term ID	P_{adj}	$-\log_{10}(P_{adj})$	T	Q	TnQ
calcium ion binding	GO:0005509	1.316×10^{-7}		721	425	46
extracellular matrix binding	GO:0050840	2.129×10^{-4}		56	425	10
platelet-derived growth factor binding	GO:0048407	3.494×10^{-2}		11	425	4

1 to 3 of 3 Page 1 of 1

GO:BP		stats				
Term name	Term ID	P_{adj}	$-\log_{10}(P_{adj})$	T	Q	TnQ
extracellular matrix organization	GO:0030198	6.808×10^{-12}		401	408	40
cell adhesion	GO:0007155	6.819×10^{-6}		1492	408	75
cell junction organization	GO:0034330	4.772×10^{-5}		722	408	42
cell differentiation	GO:0030154	6.339×10^{-5}		4350	408	147
blood vessel development	GO:0001568	4.139×10^{-3}		792	408	40
cell migration	GO:0016477	7.193×10^{-3}		1649	408	66
vasculature development	GO:0001944	1.292×10^{-2}		830	408	40
regulation of signal transduction	GO:0009966	2.166×10^{-2}		3165	408	106
cytoskeleton organization	GO:0007010	3.690×10^{-2}		1423	408	57

1 to 9 of 9 Page 1 of 1

GO:CC		stats				
Term name	Term ID	P_{adj}	$-\log_{10}(P_{adj})$	T	Q	TnQ
extracellular matrix	GO:0031012	1.478×10^{-13}		562	428	49
endoplasmic reticulum lumen	GO:0005788	1.146×10^{-5}		307	428	25
cell junction	GO:0030054	1.172×10^{-3}		2105	428	79
stress fiber	GO:0001725	4.606×10^{-2}		68	428	8

1 to 4 of 4 Page 1 of 1
